# Cognitive–behavioural therapy smartphone app for low mood and worry management in female armed forces veterans in Great Britain: protocol for a feasibility randomised controlled trial

**DOI:** 10.1136/bmjopen-2025-112494

**Published:** 2026-03-06

**Authors:** Melika Janbakhsh, Elizabeth Turnbull, Jonathan Baker, Andy Bacon, Paul Farrand

**Affiliations:** 1 Cedar Create, University of Exeter, Exeter, UK; 2 Iona Mind, Wilmington, Delaware, USA; 3 University of Chester, Chester, UK; 4 Clinical Education Development and Research (CEDAR), University of Exeter, Exeter, UK

**Keywords:** feasibility studies, digital technology, mental health, mobile applications, randomized controlled trial

## Abstract

**Introduction:**

Emotional difficulties, such as low mood and worry, are more prevalent among female forces veterans compared to their male peers. However, female veterans are more reluctant to access mental health services available for armed force veterans. To enhance help seeking, the Iona female forces veterans (IonaFFV) research app has been developed and adapted for low mood and worry management among female veterans. This feasibility randomised controlled trial primarily seeks to explore the methodological uncertainties of conducting a definitive randomised controlled trial using IonaFFV. Secondary aims seek to explore acceptability and engagement with IonaFFV. Additionally, progression criteria will be assessed to determine feasibility of moving to a definitive trial.

**Methods and analysis:**

Participants were recruited online and asked to complete two screening assessments to assess eligibility. Eligible participants were randomised using block randomisation to use either the IonaFFV or Iona sham app for 6 weeks. Recruitment and randomisation are complete, and data collection is currently ongoing. At the end of the 6-week intervention period, participants will complete the Patient Health Questionnaire-9, the Generalised Anxiety Disorder-7 and Work and Social Adjustment Scale outcome measures. At 4 weeks postintervention (10 weeks postrandomisation), participants will complete the same outcome measures in addition to the mHealth App Usability Questionnaire (MAUQ) to assess acceptability of both IonaFFV and Iona sham. At the end of the study, the participants who were in the Iona sham group will be given an option to use the IonaFFV app for 6 weeks. Proportions will be reported for feasibility and demographic data with descriptive analysis conducted for the outcome measures. Median values with IQRs will be conducted for each subscale of MAUQ.

**Ethics and dissemination:**

This study received ethical approval from the University of Exeter Psychology Ethics Committee with consent obtained from all participants. Study findings will be published in peer-reviewed journals, presented at conferences, with a final report presented to the funders of the project.

**Trial registration number:**

ISRCTN50744553.

**STRENGTHS AND LIMITATIONS OF THIS STUDY**

- This feasibility randomised controlled trial design evaluates the acceptability and implementation of the Iona female forces veterans app for female forces veterans to address mental health help-seeking barriers experienced by this group.
- Feasibility methodology and progression criteria stated to provide valuable insights to inform the design of a future definitive trial where progression criteria are met.
- Study reporting informed by SPIRIT (Standard Protocol Items: Recommendations for Interventional Trials), CONSORT (Consolidated Standards of Reporting Trials) and TIDieR (Template for Intervention Description and Replication) guidance to enhance transparency.
- The digital nature of the approach may exclude some participants who have limited access to smartphone technology, which may affect generalisability.
- Participants self-select into the study, increasing the risk of selection bias, which may limit the representativeness of the sample.

## Introduction

 Both male and female forces veterans, a person who has served in the armed forces for at least 1 day,^1^ experience barriers accessing mental health treatment.^2^ However, factors such as in-service adversity,^3^ language and male-veteran-focused branding contribute to the perception that existing services primarily reflect the needs of male veterans.^4^ Alongside limited awareness of available support options, these can create additional barriers for female veterans seeking mental health support.^5^ As a result, female veterans may feel more reluctant to access current services available for armed forces veterans and are therefore less likely to seek help.^6^ This is of significant concern given that comparisons with male veterans (16.7%; 13.5%) indicate female veterans are more likely to experience depression (25.8%) and anxiety (25.5%).^6^ Experiencing a larger treatment gap combined with a greater prevalence of common mental health difficulties therefore highlights the need to develop more appropriate and acceptable options for female forces veterans to support low mood and worry management.^5^

The structured, time-limited and goal-orientated characteristics of cognitive–behavioural therapy (CBT) make it particularly acceptable to armed forces veterans and promote engagement.^7^ Additionally, being based around a range of specific techniques supported by worksheets that empower veterans to become actively engaged in their treatment through between session work provides greater ability to adapt to the armed forces context.^8^ Previous research has indeed been undertaken to adapt CBT specifically for the armed forces veteran’s context with respect to language and imagery.^9^ Low-intensity CBT (liCBT), in which cognitive–behavioural techniques are delivered through self-help formats, has been identified as particularly acceptable for armed forces veterans.^8^ In addition to promoting self-management, its delivery via written, computer or digital platforms broadens the range of accessible support options.^10^

Growing recognition regarding the acceptability of digital technology within the armed forces community has led to an increasing number of digital mental health approaches developed specifically for this population.^11^ Developments target several areas, including the recognition of mental health difficulties and support for help-seeking behaviours,^12^ stress management,^13^ dysregulated anger^14^ and post-traumatic stress disorder^15^ .

While the use of digital mental health interventions is generally viewed positively and is being increasingly adopted within the armed forces community, several challenges remain.^11 16^ A key limitation is that the rapid expansion of digital mental health approaches currently exceeds the available research evidence needed to draw definitive conclusions about their effectiveness and to justify large-scale implementation.^17^ Additionally, although armed forces veterans are typically receptive to veteran-focused digital mental health interventions, ensuring the approaches are tailored to their specific contexts and experiences is crucial to enhance acceptability and maintain engagement.^18 19^ This is especially important for female forces veterans, who may encounter unique barriers during the transition to civilian life,^20^ including difficulties reconciling the cultural expectations of military life with their feminine identity.^21^ However, while most studies have involved veterans in the development process, participants have often been exclusively male either during the adaptation^8^ or evaluation^22^ phases.

Furthermore, previous research has failed to apply standardised intervention adaptation frameworks to guide development and therefore risk missing important dimensions necessary to maximise acceptability.^23^ Neither engaging female forces veterans in intervention adaptation nor adopting a framework that ensures adaptation dimensions of most relevance for female veterans limits the extent research findings or intervention developments can be generalised to ensure they meet their needs.^24^

Informed by Phase I (development) of the Medical Research Council (MRC) complex interventions framework,^17^ we have adapted the standalone Iona app for the management of mild to moderate low mood and worry in adults for female forces veterans.^19^ As a standalone approach, it may be particularly well suited for adoption within services currently provided for armed forces veterans, with the potential to address existing reluctance to engage.^6^ However, because it is designed to be used independently, it would not be suitable for integration within National Health Service (NHS) Talking Therapies for anxiety and depression.^25^

The app is based on liCBT techniques and uses an artificial intelligence conversational agent to promote engagement and support the user to overcome difficulties faced when engaging with the techniques.^26 27^ To guide adaptation, we worked alongside armed forces stakeholders and female forces veterans themselves and applied the ecological validity framework to explore adaptations across eight dimensions—language, persons, metaphors, content, concepts, goals, methods, context.^28^ More specifically, after consultation with a panel of female armed forces veterans and a wider armed forces stakeholder group, the armed forces-specific context, such as label and graphics, was adapted to better suit the target audience. For example, the label ‘ex-servicewomen’ was changed to ‘female armed forces veterans’ to better represent female veterans. These were done through a five-phase process.^19^ This resulted in the development of Iona for female forces veterans (IonaFFV), demonstrated to have good levels of acceptability, usability and utility.^19^

While Phase I research highlighted potential for Iona to be effective,^26 27^ an MRC Phase III randomised controlled trial (RCT) is required to reach definitive conclusions regarding effectiveness.^17^ Furthermore, conducting an RCT on IonaFFV is essential, as altering the context of existing research can threaten internal validity and may prevent replication of previously observed effects.^29^ An MRC Phase II feasibility RCT is therefore required to inform methodological, procedural and clinical outcomes.^30 31^ These are associated with (1) recruitment and retention estimates, (2) feasibility and acceptability of data collection outcome measures and procedures; (3) acceptability and usability of IonaFFV. However, beyond the presentation of means and SD, differences between study arms in clinical outcomes will not be statistically examined at this stage.^30^

## Methods and analysis

**Figure 1:**
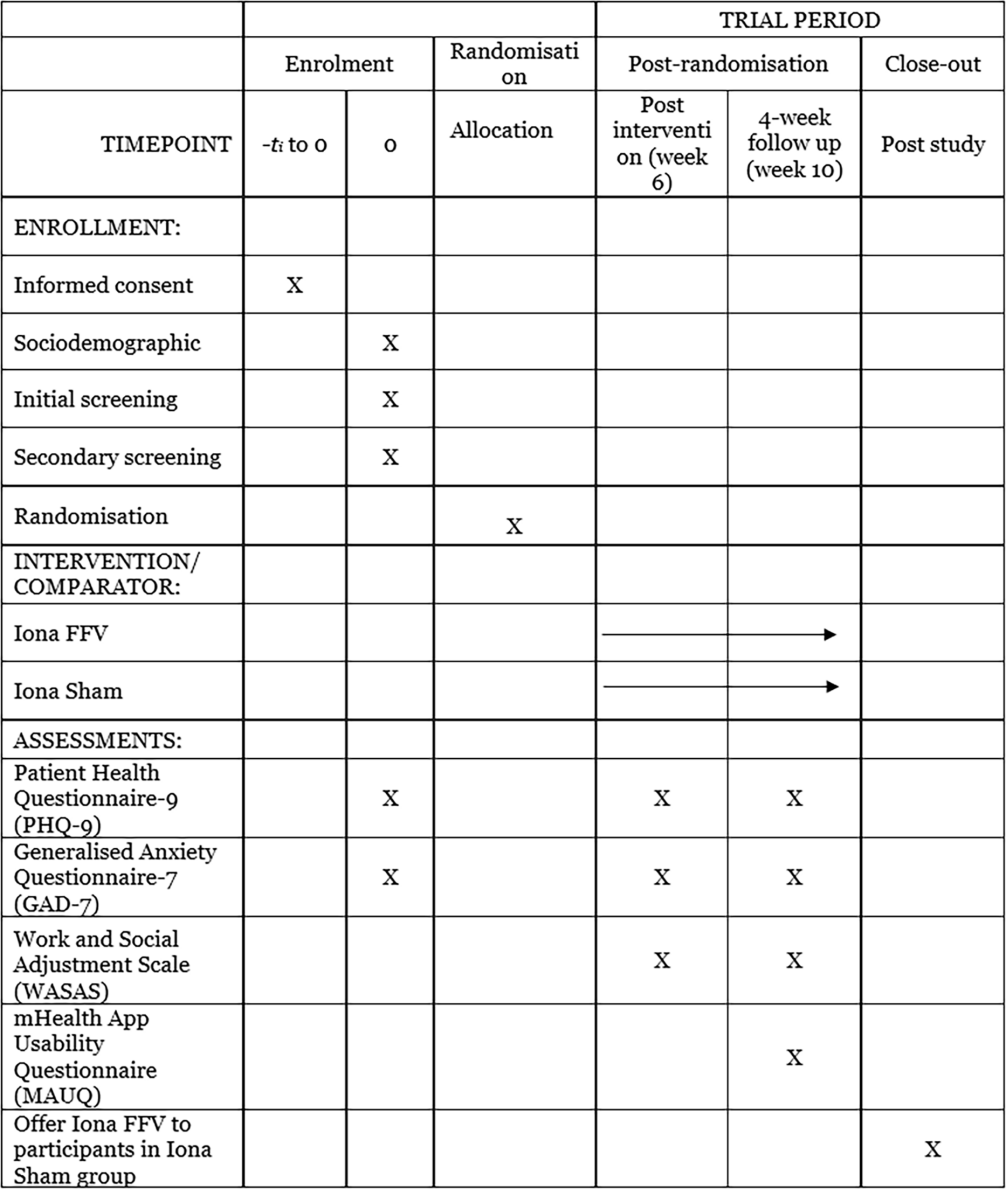
SPIRIT participants’ timeline: schedule of enrolment, interventions and assessments. IonaFFV, Iona female forces veterans; SPIRIT, Standard Protocol Items: Recommendations for Interventional Trials.

This feasibility RCT protocol (online supplemental protocol; ‘Protocol for feasibility RCT v1 230525’) follows the SPIRIT (Standard Protocol Items: Recommendations for Interventional Trials) 2025 guidelines^32 33^ (figure 1). Both the intervention and sham are reported according to the Template for Intervention Description and Replication (TIDieR)^34^ and TIDieR-Placebo^35^ checklists to enhance transparency. Furthermore, additional guidance has been adopted to report the sham to further enhance transparency.^36^

Recruitment for this study began on 4 August 2025 and ended on 22 August 2025. Data collection is ongoing, with final follow-up outcomes due on 7 November. For clarity, recruitment had concluded at the time this protocol paper was submitted. Additionally, this manuscript reflects the final protocol approved by ethics.

### Patient and public involvement

A patient and public involvement (PPI) group, comprising five female armed forces veterans and a wider armed forces stakeholder panel with expertise across the armed forces community, was involved in adapting the IonaFFV app.^19^ The wider stakeholder group met once to discuss the app’s adaptation and development, focusing on tailoring it to meet the specific needs of female forces veterans and producing an initial version. This version was subsequently reviewed in a focus group with the female armed forces to identify potential areas for improvement. Based on the feedback, further adaptations were made, and a second focus group with the same panel was convened to review the revisions.^19^

The PPI group also contributed to the design of the feasibility study protocol. Additionally, a Trial Steering Committee (TSC) comprising armed forces stakeholders and a female veteran from the adaptation process was established to work in partnership with the research team throughout the study. Furthermore, a website will be developed in collaboration with the PPI group on study completion to facilitate dissemination of the findings.

### Design and setting

This study is a double-blind feasibility RCT with intervention outcomes taken at baseline, postintervention (6 weeks) and 4-week postintervention follow-up with an additional questionnaire at the 4-week follow-up to assess usability and acceptability of the IonaFFV and Iona sham app.

Female forces veterans will engage with the IonaFFV intervention on their mobile phone in a setting with an internet or Wi-Fi signal. All research-related activities are supported through the Qualtrics platform at the University of Exeter, UK. Only data related to the feasibility objectives collected through the University of Exeter Qualtrics system will be analysed. Researchers will not have any access to the in-app data collected, such as Generalised Anxiety Disorder-7 (GAD-7) and Patient Health Questionnaire-9 (PHQ-9), and these will not be used in any data analysis. Research data collected through the University of Exeter Qualtrics will be analysed. Log data will be managed by Iona Mind and will be shared with, and analysed by, the researchers to determine engagement with the app across the two groups.

### Eligibility criteria

Participants will complete a two-stage screening process. In the first stage (ie, initial screening), they will answer general questions to establish eligibility. During this stage, participants will be included if they:

1. Identify as a female forces veteran.
2. Are at least 18 years of age.
3. Resident in Great Britain.
4. Have the ability to read and understand English.
5. Do not have a history of psychosis, mania, substance/alcohol dependence.
6. Have access to a smartphone with internet or Wi-Fi access and be able to download the app.
7. Have not started or changed antidepressant medication (ADM) in the last month.

To preserve internal validity, participants who have started or changed ADM within the past month will be excluded from the study to minimise instability of symptoms and concurrent active intervention effects.^37^

1. Not receiving mental health support during the study.

To avoid active cointervention effects, participants currently receiving mental health support will be excluded, as concurrent psychological support can influence outcomes targeted by liCBT and obscure the attribution of outcomes to the intervention.^37^

In the second stage (ie, secondary screening), participants will complete questions assessing their well-being status to determine whether the study is suitable for them. Participants will be included if:

1. PHQ-9 ≥5; <20.
2. PHQ-9–Q9 (suicide risk) ≤1.
3. GAD-7 <15.

Participants will be excluded if they do not meet the above criteria during initial and secondary screening and will be directed to sources of support if necessary.

### Digital approaches

#### IonaFFV

**Figure 2:**
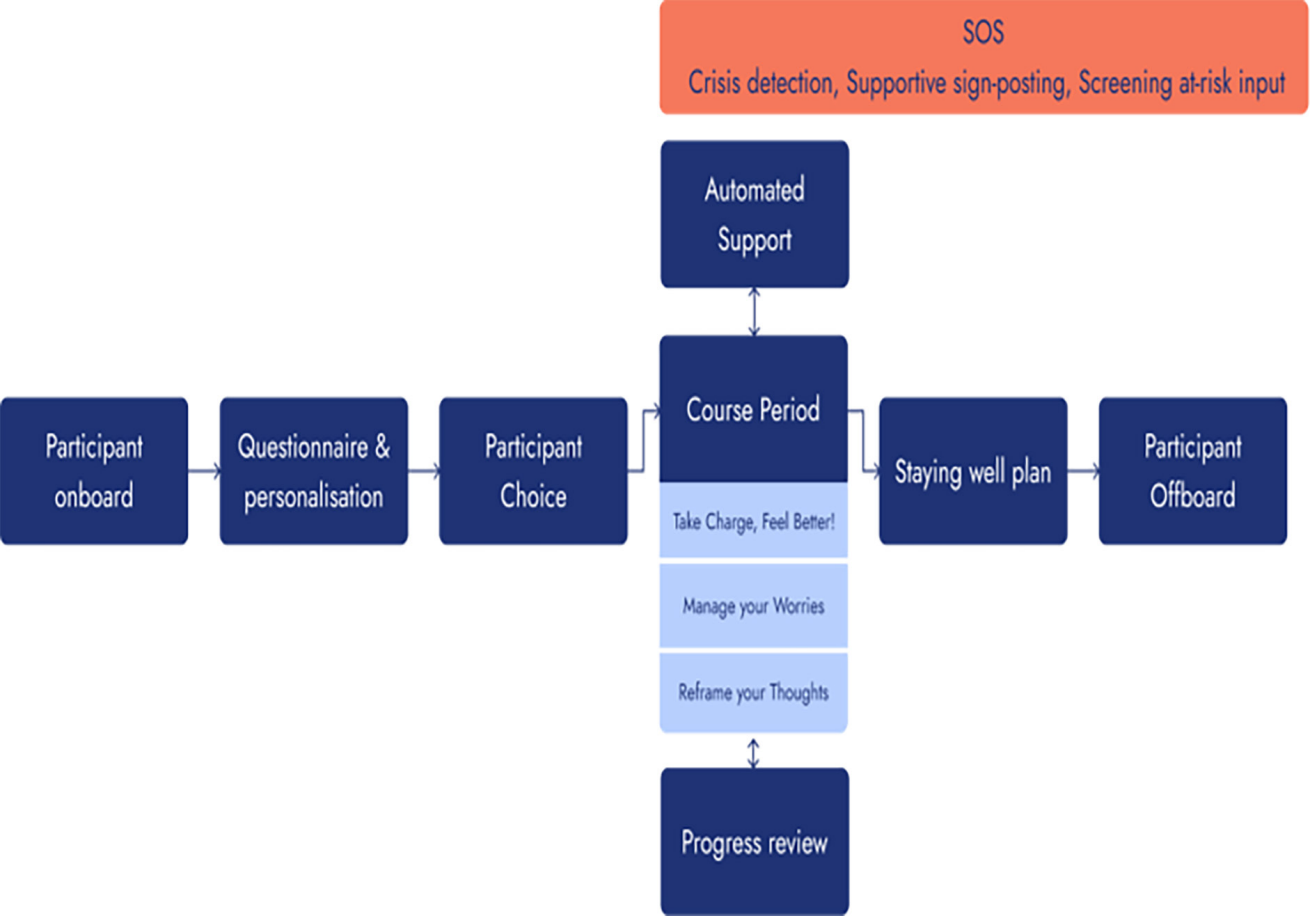
Participant progress through IonaFFV. IonaFFV, Iona female forces veterans.

IonaFFV is a native digital mobile phone-based approach to support low mood and worry management and is consistent with the characteristics associated with liCBT self-help.^10^ The app is supported by an artificial intelligence-driven chatbot without any form of human support provided. Working alongside female forces veterans and wider stakeholders, IonaFFV was adapted from the Iona app that was initially developed for the wider adult population.^19^ Participant progress through IonaFFV is presented in figure 2.

The IonaFFV app is described in accordance with the TIDieR checklist (table 1).

**Table 1:** TIDieR checklist for IonaFFV

	Brief name
**1**.	IonaFFV
	**Why**
**2**.	IonaFFV is an liCBT approach informed by specific factor techniques commonly adopted by Talking Therapies for anxiety and depression services implemented across England.^49^ All are based on evidence-based liCBT techniques to support mild-to-moderate low mood^50^ (Take Charge Feel Better), worry management (Manage Your Worries)^51^ and cognitive restructuring (Reframe Your Thoughts^52^).
	**What**
**3**.	Materials: IonaFFV is a mobile phone app based on liCBT techniques with an ‘in-app’ CA to maintain engagement with the CBT-specific factor techniques. Engagement begins with participants landing on the ‘Today’ home screen and being asked to complete the PHQ-8^53^ and GAD-7^54^ patient reported outcomes measures to assess the severity of low mood or worry. However, following the completion of these measures during initial engagement, subsequent completion is voluntary. The participant then engages with an interactive CBT Five Areas model.^55^ This serves as psychoeducation, enabling the participant to understand the nature of their low mood or worry and recognising the interaction with their thoughts, behaviours and physical feelings. The participant is then introduced to the educational modules containing the specific factor techniques informed by evidence-based protocols for behavioural activation,^56^ cognitive restructuring^57^ or worry management.^58^ All protocols are recognised by the National Institute for Health and Care Excellence.^59 60^ Detailed descriptions of the specific factors for each of these liCBT interventions as employed within IonaFFV are detailed.^61^,^63^ Each educational module includes text, illustrations and audio with the participant encouraged to engage with each module by entering raw text or selecting a predetermined response. Fundamental to CBT,^64^ following engagement with each module, participants are encouraged to apply the techniques ‘out of app’, explore outcomes arising from engagement for themselves and enter text to reflect on these.^65^
**4**.	Procedures: following download from the app store under the ‘Wellbeing’ listing, the participant is required to explicitly acknowledge the purpose of IonaFFV to support well-being and confirm they understand conditions related to the use of data collected within the app and consent to have their data processed. During engagement with the relevant educational modules containing the protocol specific factor techniques, support to help the participant overcome difficulties with the liCBT techniques is omnipresent through the CA driven by artificial intelligence. If the participant reports difficulties applying a technique, the CA will ask questions to determine the nature of the difficulty encountered and direct them to guidance to address. Furthermore, the CA monitors progress and enhances fidelity by providing proactive support if it is recognised that the participant is deviating from the protocol. Engagement with IonaFFV and specific factor techniques is further supported by behaviour change techniques promoting goal setting,^66^ with Self-Determination Theory^67^ promoting autonomy and intrinsic motivation. To maintain engagement, participants receive at least one push notification per day. These notifications include reminders to engage with scheduled activities, mark activities as completed in the app and complete modules. Throughout engagement, a progress screen presents a summary of the user’s app use and engagement with educational modules and previously entered goals. Where participants have been willing to complete the PHQ-8^53^ and GAD-7^54^ weekly within the app, scores regarding symptom severity are also presented. Furthermore, during usage, an ‘SOS’ button is available for participants to access contact details for NHS or armed forces specific emergency services. In the event the CA detects risk on the basis of raw text input into the modules, the participant will be directed to the ‘SOS’ button, provided with the signposting information and reminded that IonaFFV is not intended to deliver treatment and should not be used outside of the context of a well-being self-help aid.
	**Who provided**
**5**.	No human support is required for the approach. Engagement with the specific factor techniques is supported through an in-app artificial intelligence CA.
	**How**
**6**.	The approach is delivered using a smartphone app with internet access and Wi-Fi access.
	**Where**
**7**.	As long as there is internet or Wi-Fi connectivity, participants are able to engage with IonaFFV at a time and location of their choosing.
	**When and how much**
**8**.	Participants can engage with IonaFFV over a 6-week duration at times of their choosing with no limits placed on the number, or duration, of engagement sessions. Advice within IonaFFV is, however, provided to propose that a higher number of engagement sessions of limited duration is better than less engagement with sessions of longer duration. To be considered an ‘engaged user’ each participant is required to complete at least two educational modules and a minimum of two sessions within the 6-week intervention duration.^26 27^
	**Tailoring**
**9**.	To represent the preferences of female forces veterans and wider armed forces context, IonaFFV is an adaptation of the Iona app^26 27^ for the management of mild-to-moderate low mood or worry. Adaptation was informed by the application of the EVF,^68^ a mixed methods study comprising an armed forces stakeholder group and focus group comprising female forces veterans, with the MAUQ^44^ used to determine acceptability, usability and usefulness.^19^
	**Modifications**
**10.**	None
	**How well**
**11**.	Planned analysis of log data^69^ will examine progress of participants through IonaFFV. Analysis will record engagement with respect to monitoring number of sessions completed, session duration, with engagement determined by completion of specific techniques.^70^

CA, conversational agent; CBT, cognitive–behavioural therapy; EVF, ecological validity framework; GAD-7, Generalised Anxiety Disorder-7; IonaFFV, Iona female forces veterans; liCBT, low-intensity CBT; MAUQ, mHealth App Usability Questionnaire; N/A, not applicable; NHS, National Health Service; PHQ-8, Patient Health Questionnaire-8; TIDieR, Template for Intervention Description and Replication.

#### Iona sham

**Figure 3:**
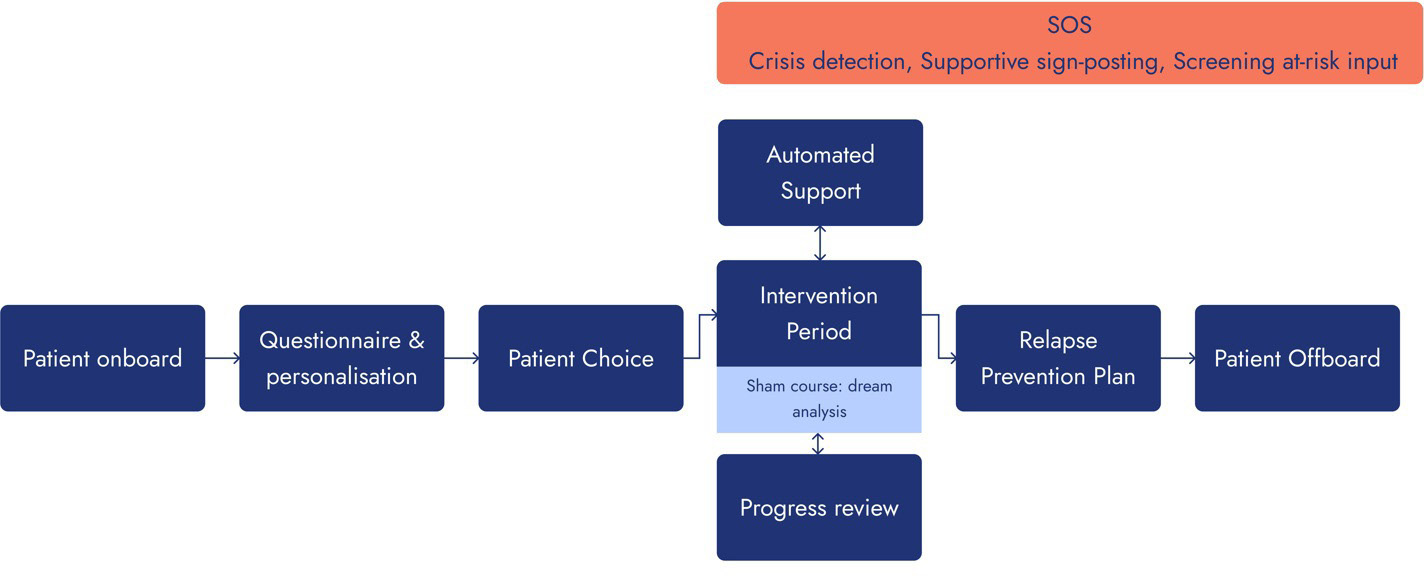
Participant progress through Iona sham.

The Iona sham is located on a mobile phone app and designed to mimic the appearance of IonaFFV. To give the impression of being an active intervention to support low mood and worry management, the app includes background information on dream analysis and supports the participant to progress through ‘meditation-like’ exercises (figure 3). Prior to use, the sham was beta tested but not validated.^19^ In an attempt to engage participants for approximately the same amount of time as experienced with IonaFFV, participants are encouraged to interact and engage with activities contained within the sham app.

The Iona sham is described in accordance with the TIDieR checklist (table 2).

**Table 2:** TIDieR-placebo checklist for Iona sham

	Brief name
**1**.	Iona sham
	**Why**
**2**.	The Iona sham was developed and implemented with recognition that there is currently no consensus as to what is considered a digital sham to fully guide development.^36^ To eliminate the potential for the name to influence effectiveness, the name of the app was the same as the intervention.^36^ The nature of the sham was to explore the potential for IonaFFV to be effective for potential subsequent evaluation at a later research phase.
	**What**
**3**.	Materials: the Iona sham is an unsupported mobile phone app adapted for female forces veterans that has been developed and delivered on the same app infrastructure to ensure a consistent look and usability to IonaFFV. Engagement starts when the participants access the ‘Today’ home screen where they are asked to complete the PHQ-8^53^ and GAD-7,^54^ with subsequent completion voluntary following initial completion. The participant then progresses to each of the six modules that discuss dream analysis^71^ and instructions to undertake ‘meditation-like’ exercises. Participants are encouraged to engage with each of the modules by either entering text into a dream diary to describe their dreams or following simple instructions to support meditation, with modules enhanced through text, illustrations and audio. The sham, however, did not include any specific factor information associated with analysing dreams, with efforts made to ensure the ‘meditation-like’ exercises are not recognised as evidence-based by the National Institute for Health and Care Excellence.
**4**.	Procedures: the participant is required to acknowledge the purpose of the sham app to support well-being, complete consent and confirm conditions regarding data use following download. Once completed, participants are taken to the ‘Today’ screen where they are presented with six modules to work through. Engagement begins with participants landing on the Today home screen, after which they are introduced to descriptions of approaches such as Dream Analysis,^71^ but no specific factor content enabling the approaches to be applied. They then progress through simple instructions to engage in ‘meditation-like’ lessons. To maintain engagement, participants are sent at least one push notification per day. These are intended to remind the participant to engage with the module content and record completed activities which inform a progress screen to summarise completed modules. If participants have been completing the PHQ-8^53^ and GAD-7^54^ weekly within the app, symptom severity scores are also presented. Support for risk management is provided through an ‘SOS’ presented on each screen that provides contact details for NHS or armed forces specific emergency services.
	**Who provided**
**5**.	No human support of any type is available.
	**How**
**6**.	The intervention is delivered using a smartphone app with internet and Wi-Fi access.
	**Where**
**7**.	As long as there is internet or Wi-Fi connectivity, participants are able to engage with the sham at a time and location of their choosing.
	**When and how much**
**8**.	Participants can engage with the Iona sham for up to 6 weeks, choosing where, when and for how long they engage. However, advice is given to them that they may find engaging more often with briefer sessions preferable. To be considered an ‘engaged user’ each participant is required to complete at least two modules and a minimum of two sessions.
	**Tailoring**
**9**.	The Iona sham app will be delivered within the same app framework using approximately the same app and cloud infrastructure as IonaFFV to ensure a consistent look and feel for female forces veterans and wider user experience.
	**Modifications**
**10.**	None
	**How well**
**11**.	Analysis of log-data^69^ recording engagement, number of sessions completed and session duration will be undertaken.

GAD-7, Generalised Anxiety Disorder-7; IonaFFV, Iona female forces veterans; N/A, not applicable; NHS, National Health Service; PHQ-8, Patient Health Questionnaire-8; TIDieR, Template for Intervention Description and Replication.

#### IonaFFV and Iona sham app interfaces

**Figure 4:**
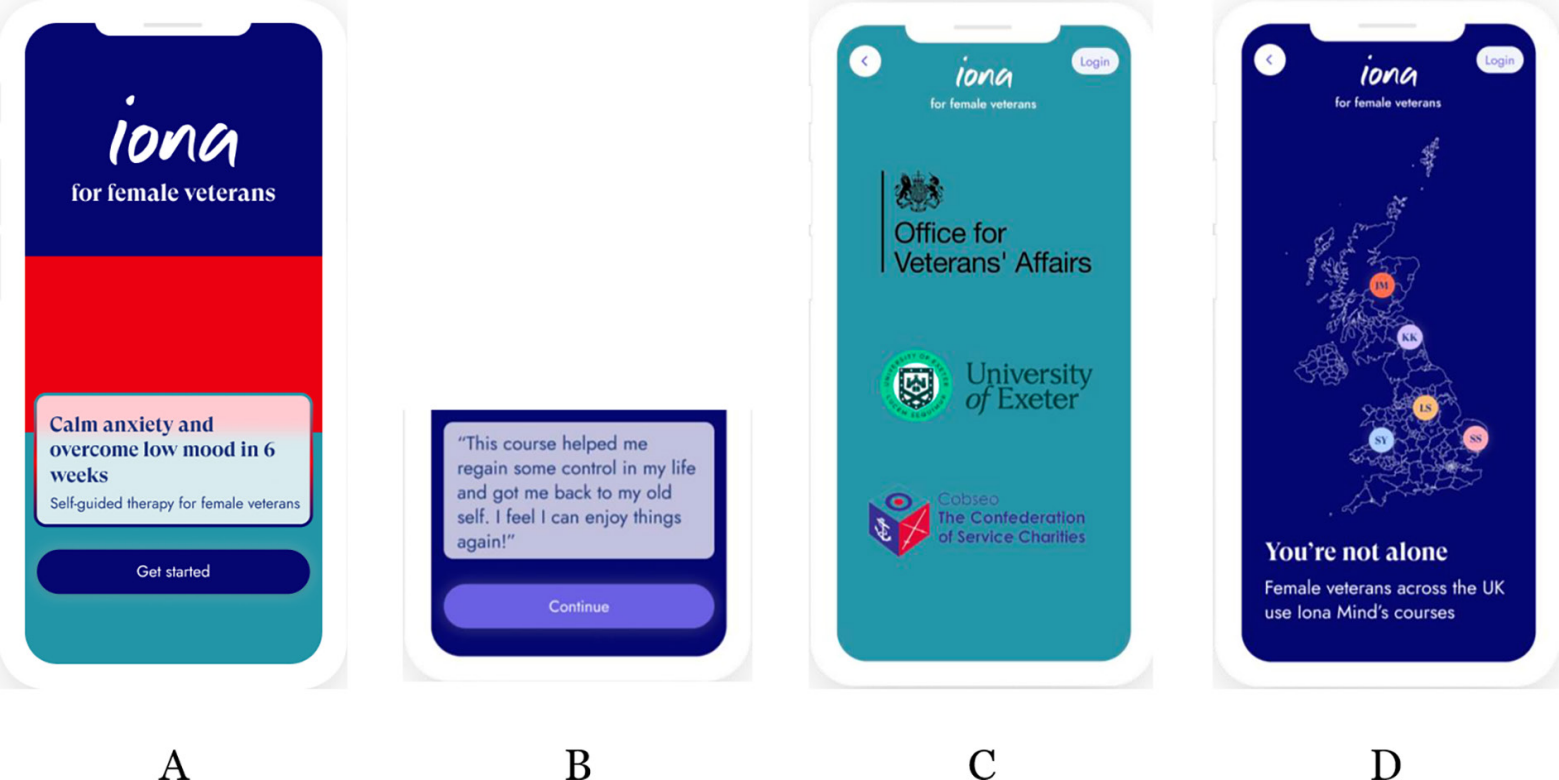
Screenshots of IonaFFV and Iona sham app. IonaFFV, Iona female forces veterans.

Both IonaFFV and sham app share general features in their interface. Figure 3 captures some of the Iona app’s interface features, which are representative of some of the app’s features that were adapted during the development process.^19^ Iona app graphics use the tri-service colours to emphasise the association with the armed forces (figure 4A). Additionally, at the start of the app, several quotes are shown. These quotes are a mixture of those from female forces veterans as well as more generally acknowledging the transition from service in the armed forces to civilian life (figure 4B). Further, logo and names of trusted organisations are provided at the start of the app to enhance perceived credibility and expectancy of effectiveness of the app (figure 4C). Finally, a map is provided in the app to indicate other users’ location, which enhances engagement with the app (figure 4D).

### Outcome measures

#### Primary outcome measures

##### Feasibility outcomes

Feasibility outcomes (table 3) are focused on methodological, procedural and clinical uncertainties.^31^ These examine recruitment rates, eligibility criteria, data collection and completion and attrition. Uncertainties related to IonaFFV and Iona sham include participant adherence, acceptability and usability.

**Table 3:** Overview of feasibility outcome and progression criteria

Outcome	Evaluation	Progression criteria
Recruitment and eligibility	Percentage assessed for eligibility that fulfil inclusion criteria and included. Participants expressed ambiguity regarding eligibility criteria. Reasons for ineligibility. Reasons for non-participation.	≥90% interested in participating No criteria set No criteria set No criteria set
Data collection	Percentage of participants completing clinical outcome measures at all assessment periods. Number of missing items.	≥70% ≤10% per questionnaire
Study attrition	Rates of study drop-out.	≤30%
Intervention engagement	Rates app downloaded. Rates app used after download. Considered an engaged user by completing at least two educational modules and a minimum of two sessions. The difference considered an engaged user between IonaFFV and Iona sham. Number of modules accessed.	≥40% ≥15% ≥20% ≤30% No criteria set
Intervention acceptability and usability	Percentage of participants strongly/agree with questions related to ‘Interface and Satisfaction’. Percentage of participants strongly/agree with questions related to ‘Ease of Use’.	≥70% ≥70%
Success of blinding	Percentage of participants correctly identifying Iona sham app as the sham.	≤20%

IonaFFV, Iona female forces veterans.

##### Progression criteria

Feasibility outcomes are presented alongside respective progression criteria to inform decision making^38^ regarding progression to an MRC Phase II feasibility randomised controlled.^17^ Methodological and procedural progression criteria are informed by a similar research study examining an online CBT intervention for depression and anxiety.^39^ Clinical progression criteria are informed by a clinical review examining engagement with direct-to-consumer mental health apps.^40^

These thresholds for progression criteria are set as feasibility objectives rather than as clinical assumptions or a measure of success or effectiveness of the intervention. Recent methodological guidance on progression criteria for an external feasibility pilot trial recommends that thresholds need to be interpreted as guidelines rather than prescriptive rules and be context-specific and aligned with feasibility objectives.^38^

This is particularly important given there are no universally accepted engagement benchmarks for engagement, and that engagement with mental health and app in real-world settings is generally low, with the majority of users disengaging after downloading the app.^40^ Evidence from a systematic review of real-world engagement with digital self-help intervention indicated that between 21% and 88% of users achieved at least minimal engagement defined as using the intervention at least once or completing one module or one assessment.^41^

Therefore, for the purposes of this study, an engaged user is defined as a user who completes at least two educational modules (ie, lessons) and a minimum of two sessions during the 6-week period of using the Iona app. Specifying a minimum engagement of two lessons and two sessions is consistent with existing literature suggesting that brief or partial exposure is common and may be sufficient for feasibility assessment in early stage digital interventions.

Further, although there is no universally agreed threshold to define an acceptable mHealth App Usability Questionnaire (MAUQ) score, responses of strongly agree, agree and somewhat agree are considered acceptable by some researchers.^42 43^

### Secondary outcome measures

#### Sociodemographic variables

Sociodemographic data will be collected from participants and include age range, gender, ethnicity group, service, rank at discharge, year left service, length of service, whether discharge was planned, reason for unplanned discharge and whether they were deployed on operation.

#### Psychological outcomes

Psychological outcomes include the PHQ-9 to examine the severity of low mood and the GAD-7 for worry. These measures are completed at baseline, postintervention (week 6) and 4-week follow-up (week 10). The Work and Social Adjustment Scale (WASAS) is used to assess functional impairment and the impact of mental health difficulties on daily life, including work, home and social activities. WASAS is collected at postintervention (week 6) and 4-week follow-up (week 10). These measures are collected by the researchers and are separate from those completed within the IonaFFV and Iona sham app.

#### Acceptability and Usability

To understand the acceptability of the IonaFFV and Iona sham, all participants will complete the MAUQ^44^ at week 10, to evaluate usability, ease of use and overall satisfaction with the app.

### Study sample and recruitment

Informed by sample size recommendations to assess feasibility outcomes of sample and estimate sample size for an MRC Phase III RCT,^17^ we aim to recruit a sample of 60 female forces veterans.^45^ With a total sample size of 60, a 70% retention rate at the 4-week follow-up can be estimated with a margin of error of approximately ±12% using a 95% CI or ±10% using a 90% CI.

The study is being conducted online on University of Exeter’s Qualtrics account. This platform hosts the participant information sheet, consent form, demographic questionnaire, screening measures and outcome measures which will be administered at 6 and 10 weeks postrandomisation.

Recruitment is primarily conducted online through public and private armed forces community Facebook groups, armed forces veterans’ charity and community sector organisations’ social media posts, and armed forces social media platforms, such as LinkedIn and Facebook, and word of mouth. Armed forces Facebook pages will be contacted to seek permission to post the study flyer on their page.

Interested individuals will click a link or scan a QR code that directs them to the study on Qualtrics, where they can access and download the participant information sheet. The sheet outlines the study aims, participation requirements, data storage and data management processes, and withdrawal information. If a participant withdraws, they are not required to submit any further data. Researchers’ contact details will be provided for further clarification. Additionally, the study inclusion criteria and randomisation process will be clearly stated.

On signing the consent form, participants are directed to the sociodemographic and first screening questionnaire which includes questions about age range, gender identification, service branch, rank, etc. Eligible participants are then asked to complete the second screening which includes the PHQ-9 and GAD-7 to determine study suitability. Participants who do not meet the inclusion criteria are screened out and signposted to appropriate resources. Those who meet the inclusion criteria will be asked to provide their email address and phone number to be contacted for study entry.

At this point, participants receive electronic confirmation of their inclusion in the study, along with a unique trial number. Informed by the randomisation scheme, participants will be given a link with instructions on how to download the Iona app on Android or iOS devices. All participants are asked to download the same app. The app is available under the ‘Wellbeing’ listing on Apple Store and Google Play to download. To log in and use the app, participants will be asked to enter their unique code which then brings them to either the IonaFFV or the Iona sham version of the app, whichever one they are randomised to. Contact details for the study researchers are also provided in case participants require further assistance or experience difficulties downloading the app.

### Randomisation and allocation concealment

Randomisation involves assigning participants to use the IonaFFV app or using the Iona sham app. Participants are given a unique code from either group A or group B, which corresponds to the IonaFFV app or the Iona sham app. Neither the researchers nor the participants are aware of which group contained the codes for which app. Block randomisation with a fixed block size of three will be used to ensure balanced group sizes throughout the recruitment period. Randomisation continues until 60 participants (30 in each arm) are randomised, or until the recruitment deadline is reached, whichever occurs first. Each participant’s allocation and randomisation date will be recorded. The trial is conducted with an exploratory framework. Randomisation will be conducted by researchers at the University of Exeter using allocation codes generated by the Iona system. This process ensures concealment of the allocation sequence to minimise selection bias and confounding.

Following randomisation, participants will be sent a link to download the app. Once the app is downloaded, participants will be asked to input their unique code. Codes from one group will open the IonaFFV version of the app onto a participant’s phone, and codes from the other group will open the Iona sham app.

Participants will be encouraged to download the app as soon as possible. An automated sequence of emails on day 2, 14 and 28 postrandomisation will be sent to remind participants to download the app and reiterate instructions.

This study employs a double-blind design, in which neither participants nor researchers are aware of the allocation assignments. PF will not be involved in any data collection or data analysis. Data collection and analysis will be solely done by the study researchers (MJ and ET).

Accidental unblinding may occur if a member of the research team receives communication from a participant that reveals their intervention allocation. In such cases, the researcher will inform the wider team and will be excluded from any data analysis to preserve integrity of the blinding process. Unblinding will occur only after the completion of all data analyses by researchers from the study team. At that stage, participants allocated to the Iona sham group will be offered access to the IonaFFV app for a 6-week period outside the study.

### Trial management and data collection

The trial is overseen by a trial management group led by the chief investigator and lead researcher, who are responsible for the day-to-day conduct, data monitoring, protocol adherence and safety reporting. This is alongside the TSC who provide independent oversight. As the trial sponsor, the University Sponsorship Committee ensures compliance with General Data Protection Regulation, Good Clinical Practice and ethics approvals. All amendments to the protocols are logged and reported to the TSC and trial sponsor, and where substantive changes are required, to the ethics committee.

Data are being collected online through questionnaires on the Qualtrics platform. Participants receive an automated email at 6 and 10 weeks postrandomisation inviting them to complete the follow-up questionnaires. Participants are required to enter their unique trial number before completing the questionnaires in order to link their responses. Personally identifiable data and a spreadsheet containing the link codes are stored separately from the rest of the data on an encrypted Excel spreadsheet stored on a secure password protected server. Only researchers directly involved in data collection and analysis have access to this data. Personal information will be stored separately to participant data, via a password-protected university computer. Data will be pseudonymised, using a five-digit code to identify participants and stored separately to personal information.

An initial meeting with the independent Data Monitoring Committee (DMC) established its role and the frequency of reporting during recruitment and follow-up. The Chair receives weekly updates on study progress and adverse events (AEs) and convenes meetings with other DMC members, including a female forces officer, an NHS veterans service lead and a regional commissioner. Prior to unblinding, the DMC will review a draft results section and aggregated data to ensure compatibility.

#### Adverse events

For the purposes of this study, an AE is defined as any deterioration in a participant’s mental state or behaviour following the administration of the approach. This includes events that may not be directly caused by or related to the intervention. An AE is flagged when a participant’s score on item 9 (ie, suicidal ideation screening) of the PHQ-9 increases to 2 (more than half the days) or 3 (nearly every day) or the participant’s overall PHQ-9 deteriorates from ‘mild’ or ‘moderate’ to ‘severe’ during the week 6 and week 10 follow-ups. AEs will be monitored and recorded by the research team (MJ and ET) within 1 working day, after which they will provide follow-up information as soon as possible.

#### Adverse reaction

An adverse reaction (AR) is defined similarly to an AE; however, it requires establishing a reasonable possibility that the event is related to the app.

#### Responsibilities

PF, as the chief investigator, is responsible for reporting to the TSC for consideration when an event meets criteria for an AR, and for disseminating to the sponsor and ethics committee where the AE is considered to represent a potential AR.

#### Serious adverse events

Recording serious AE is not possible in this study, as no mental-health or community-based services are involved.

### Analysis

The results of this study will be analysed and reported in accordance with the Consolidated Standards of Reporting Trials (CONSORT) 2025 guidelines for randomised feasibility trials.^30^ Analysis will be descriptive and address the outcomes relating to the feasibility of the study procedures and intervention engagement. Progression criteria will determine decisions regarding progression to an MRC Phase II pilot RCT.^17^ All participants who meet the minimum dose will be included within the analysis. Missing data will be reported descriptively. Given the feasibility nature of the study, no imputation will be performed. However, patterns of missingness will be examined to inform the design of a future pilot trial.

#### Sociodemographic variables

Proportions will be reported for sociodemographic variables.

#### Psychological outcomes

Descriptive analyses of means and SD will be conducted for the PHQ-9, GAD-7 and WASAS at each study time point. Prior to unblinding, the DMC will review a draft of the results section that employs coded identifiers to represent each trial arm. This review will evaluate the accuracy, clarity and consistency of data reporting, with study researchers only becoming unblinded once the draft has been reviewed and approved by the DMC.

#### Acceptability and usability

Median values with IQR will be calculated for each subscale of the MAUQ subscales.^43^ Additionally, each subscale will be analysed to determine the percentage of agreement with each statement.^43^

## Ethics and dissemination

The study has been approved by the University of Exeter Psychology Ethics Committee (ref. 10130867) and adopted by the University Sponsorship Committee. To ensure the welfare and rights of all participants, it will be conducted in accordance with the 2024 revision of the Declaration of Helsinki.^46^ Participants will be provided with a detailed participants information sheet. Consent will be obtained from all participants. All research data will be handled according to General Data Protection Regulation ((Council regulation) 2016/679)^47^ with data collected via Qualtrics stored on secure servers at the University of Exeter.

Ethical approval also addressed participant engagement within IonaFFV as a digital well-being research tool. Following the download, participants are presented with three mandatory consent screens, each of which must be acknowledged by consenting before being able to proceed with the standard sign-up process and gain full access to the app. The first screen explains that the app is a research tool and that no human will be monitoring participants’ engagement. It also stresses that the app is not developed for people who consider themselves at risk, and in this event, they are instructed to contact emergency services on details provided. The second screen provides general consent for data storage and processing, stating that the app will store the information entered within the app and that the data may be reviewed to improve the app. Finally, the third screen specifies that certain information may be shared with the University of Exeter for research purposes. It clarifies that while research findings may be published, participants’ identities will remain anonymous. Study findings will be published in a report to the funder, open-access journal, conference presentations and via stakeholder groups’ dissemination channels.

Findings from this trial will be disseminated in line with the latest CONSORT 2025 reporting guidelines.^48^ They will be shared through a peer-reviewed publication, conference presentations and a detailed written and oral report to the study funders, Office for Veterans’ Affairs.

## Discussion

There is a treatment gap between male and female forces veterans, with female veterans experiencing a higher prevalence of depression and anxiety.^40^ However, previous research has largely failed to engage female forces veterans in intervention adaptation or apply relevant adaptation frameworks, limiting the generalisability of findings to their needs.^24^

This paper presents the study protocol for a Phase II feasibility RCT examining a smartphone-based CBT intervention to support low mood and worry management among female forces veterans in Great Britain. Results have the potential to contribute to a better understanding regarding the potential effectiveness of the IonaFFV intervention in improving female forces veterans’ mental health.

Should the progression criteria be met, results may be used to inform the design of a Phase II pilot RCT. Insights from the pilot could then guide modifications to design improvements for a Phase III definitive trial to evaluate the intervention’s effectiveness in improving low mood and reducing worry among female forces veterans. If progression criteria are not met, the intervention and design of the feasibility study could be revised to address identified limitations prior to further evaluation.

### Trial sponsor

University of Exeter, sponsor representative: Suzy Wignall, res-sponsor@exeter.ac.uk. The trial sponsor (University) and funder (Office for Veterans’ Affairs) have no role in conducting, analysing or reporting of the trial. This paper has been produced with funding from the Office for Veterans' Affairs in April 2023 as part of an independent research initiative. The views, findings, and conclusions expressed herein are those of the authors and do not reflect the policies or positions of the UK Government. The funder, however, has to approve the paper and its contents prior to publication.

Data, trial protocol and statistical analysis plans are available upon request by contacting the lead author, MJ, mj268@exeter.ac.uk.

## Supplementary material

10.1136/bmjopen-2025-112494 online supplemental file 1
