# Supplementary material for: Cognitive–behavioural therapy smartphone app for low mood and worry management in female armed forces veterans in Great Britain: protocol for a feasibility randomised controlled trial
Source: BMJ Open. 2026 Mar 6;16(3):e112494. doi: 10.1136/bmjopen-2025-112494 (PMC12970052; doi:10.1136/bmjopen-2025-112494)

**Protocol for a feasibility evaluation of a smartphone based Cognitive Behavioural Therapy (CBT) intervention for the treatment of low mood or support for worry management in female armed forces veterans in England, Wales, and Scotland.**

**Trial Protocol Version 1.0 dated 23/05/2025**

**Funded by: Defence and Security Accelerator Programme; Veterans health Innovation Fund**

|  |  |
| --- | --- |

**RESEARCH REFERENCE NUMBERS**

| **IRAS number:**  **ISRCTN:** | **335603**  **ISRCTN50744553** |
| --- | --- |

###

###

###

###

###

### SIGNATURE PAGE

The undersigned confirm that the following protocol has been agreed and accepted and that the Chief Investigator agrees to conduct the trial in compliance with the approved protocol and will adhere to the principles outlined including clinical trial regulations, GCP guidelines, the Sponsor’s SOPs, and other regulatory requirements.

I agree to ensure that the confidential information contained in this document will not be used for any other purpose other than the evaluation or conduct of the clinical investigation without the prior written consent of the Sponsor.

I also confirm that I will make the findings of the trial publicly available through publication or other dissemination tools without any unnecessary delay and that an honest accurate and transparent account of the trial will be given; and that any discrepancies and serious breaches of GCP from the trial as planned in this protocol will be explained.

| **For and on behalf of the Trial Sponsor:** | | |
| --- | --- | --- |
| Signature: 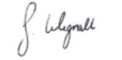 |  | Date: 02/01/2025 |
| Name (please print): Suzy Wignall  ...................................................................................................... |  |  |
| Position: Senior Clinical Research Governance Manager  ................................................................................ |  |  |
| **Chief Investigator**  **Signature:** 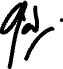 |  |  |
| **Date: 15/10/25**  **Name: Professor Paul Farrand** | | |

### KEY TRIAL CONTACTS AND GENERAL STUDY BACKGROUND

**Table 3 Key contacts**

| Chief Investigator | Professor Paul Farrand, Professor of Evidence Based Psychological Practice, Sir Henry Wellcome Building for Mood Disorders Research; Clinical Education, Development and Research (CEDAR), Psychology; Faculty of Health and Life Sciences; University of Exeter, EX4 4QG. |
| --- | --- |
| Co-Investigator | Professor Andy Bacon, Professor of Global and Armed Forces Health, University of Chester, CH1 4BJ. Previous NHS England & NHS Improvement, Ex-Head of Policy & Strategy for Armed Forces. |
| Lead Researcher | Dr Melika Janbakhsh, Postdoctoral Research Associate, Washington Singer, Cedar Create; Psychology; Faculty of Health and Life Sciences; University of Exeter, EX4 4QG. |
| Trial manager | Professor Paul Farrand (as above) |
| Sponsor | University of Exeter, Sponsor Representative: Suzy Wignall. |
| Funder(s) | Defence and Security Accelerator Programme; Veterans Health Innovation Fund |
| Clinical Trials Unit | Feasibility RCT (Not adopted) |
| Key Protocol Contributors | Paul Farrand  Andy Bacon  Jonathan Baker (Iona Mind)  Melika Janbakhsh (lead researcher) |
| Statistician | None (Melika Janbakhsh as Lead Researcher) |
| Site Team | All recruitment is online only through potential participants following a link to the University of Exeter study web site |

Conflicts of Interest: JB is a full-time employee, and PF was on a part time paid sabbatical with Iona Mind Inc from the University of Exeter ending October 1^st^ 2025. All other authors have confirmed they have no conflicts of interest to declare.

The trial sponsor (University) and funder (Office for Veterans Affairs) have no role in conducting, analysing or reporting of the trial. The funder however has to approve the paper and its contents prior to publication.

**Table 5 LIST of CONTENTS**

[SIGNATURE PAGE 2](#_Toc220919773)

[KEY TRIAL CONTACTS AND GENERAL STUDY BACKGROUND 3](#_Toc220919774)

[1 BACKGROUND 8](#_Toc220919775)

[2 RESEARCH QUESTION 9](#_Toc220919776)

[3 STUDY OBJECTIVE 9](#_Toc220919777)

[4 KEY WORDS 9](#_Toc220919778)

[5 STUDY SUMMARY 10](#_Toc220919779)

[6 TRIAL MANAGEMENT/RESPONSIBILITIES 11](#_Toc220919780)

[6.1 Participant registration/randomisation procedure 11](#_Toc220919781)

[6.2 Data management 11](#_Toc220919782)

[6.2.2 Data Management Plan 12](#_Toc220919783)

[6.2.3 Data format and types 12](#_Toc220919784)

[6.2.4 Data exploitation 12](#_Toc220919785)

[6.3 Data protection/confidentiality 12](#_Toc220919786)

[6.3.1. Data protection 12](#_Toc220919787)

[6.3.2 Digital Information 13](#_Toc220919788)

[6.3.3. Participant data 13](#_Toc220919789)

[6.3.4 Responsibility 14](#_Toc220919790)

[6.3.5 Data monitoring 14](#_Toc220919791)

[6.3.5a Data monitoring committee 14](#_Toc220919792)

[6.3.6 Breach of confidentiality 14](#_Toc220919793)

[6.4 Trial documentation and archiving 14](#_Toc220919794)

[6.5 Authorisation of Participating Site 15](#_Toc220919795)

[7. PARTICIPANT INCLUSION/EXCLUSION CRITERIA 15](#_Toc220919796)

[8. TRIAL PROCEDURES 15](#_Toc220919797)

[8.1 Participant identification 15](#_Toc220919798)

[8.2 Screening 16](#_Toc220919799)

[8.3 Consent 16](#_Toc220919800)

[8.4 Randomisation scheme 16](#_Toc220919801)

[8.5 Protection from bias 16](#_Toc220919802)

[8.6 Method of implementing the randomisation/allocation sequence 17](#_Toc220919803)

[8.7 Blinding 17](#_Toc220919804)

[8.8 Baseline data 17](#_Toc220919805)

[8.9 Trial assessments 17](#_Toc220919806)

[8.10 Long term follow-up assessments 19](#_Toc220919807)

[8.11 Payment 19](#_Toc220919808)

[8.12 Withdrawal criteria 19](#_Toc220919809)

[8.13 End of trial 19](#_Toc220919810)

[8.14 Assessment of engagement at 6-week treatment end 19](#_Toc220919811)

[9 INTERVENTIONS 20](#_Toc220919812)

[9.1 Iona mobile phone app adapted for Female veterans (IonaFFV) 20](#_Toc220919813)

[9.2 SHAM control app 21](#_Toc220919814)

[10 OUTCOME MEASURES/ENDPOINT 22](#_Toc220919815)

[10.1 Demographic Data 22](#_Toc220919816)

[10.2 Outcome measures 22](#_Toc220919817)

[10.3 Timeframe for administration of demographic and outcome measures 25](#_Toc220919818)

[10.4 Primary endpoint/outcome 25](#_Toc220919819)

[10.5 Secondary endpoints 25](#_Toc220919820)

[10.6 Exploratory endpoints 25](#_Toc220919821)

[10.7 Feasibility outcomes 26](#_Toc220919822)

[11. STATISTICS AND DATA ANALYSIS 26](#_Toc220919823)

[11.1 Sample size calculation 26](#_Toc220919824)

[11.2 Planned recruitment rate 26](#_Toc220919825)

[11.3 Statistical analysis plan 26](#_Toc220919826)

[11.4 Summary of baseline data and flow of patients 26](#_Toc220919827)

[11.5 Primary analysis 27](#_Toc220919828)

[11.6 Secondary analyses 27](#_Toc220919829)

[11.7 Outcome reporting 27](#_Toc220919830)

[11.8 Interim analysis and criteria for the premature termination of the trial 27](#_Toc220919831)

[11.9 Other statistical considerations 27](#_Toc220919832)

[12. ADVERSE EVENTS 28](#_Toc220919833)

[12.1 Participant welfare and safety 28](#_Toc220919834)

[12.2 Risk management processes 28](#_Toc220919835)

[12.3 Definitions 28](#_Toc220919836)

[12.4 Recording and reporting of AEs and ARs 29](#_Toc220919837)

[12.5 Responsibilities 30](#_Toc220919838)

[12.6 Reporting urgent safety measures 30](#_Toc220919839)

[13. RISK MANAGEMENT 31](#_Toc220919840)

[13.1 Risk assessment and reporting 31](#_Toc220919841)

[13.1.1 Risk management during baseline assessment 31](#_Toc220919842)

[13.1.2 Risk management in trial 31](#_Toc220919843)

[13.2 Preventing abuse of participants and risk analysis 31](#_Toc220919844)

[13.3 Identifying suicide risk 32](#_Toc220919845)

[13.4 University of Exeter Qualtrics page at second screening (as baseline), 6 and 10 week follow up 32](#_Toc220919846)

[13.5 For participants reporting significant levels of depression at any of the follow-up assessments (defined as PHQ-9 score >20) 34](#_Toc220919847)

[13.6 Exclusions- Bipolar and Psychosis 36](#_Toc220919848)

[14. DISSEMINATION POLICY 38](#_Toc220919849)

[15. REFERENCES 39](#_Toc220919850)

[APPENDIX 40](#_Toc220919851)

**Table 6 LIST OF ABBREVIATIONS**

| **Abbreviation** | **Full term** |
| --- | --- |
| ADM | Antidepressant Medication |
| AE | Adverse Event |
| AES | Advanced Encryption Standard |
| AI | Artificial Intelligence |
| AR | Adverse Reaction |
| CI | Chief Investigator |
| CBT | Cognitive Behavioural Therapy |
| CRF | Case Report Form |
| CRO | Contract Research Organisation |
| DMC | Data Monitoring Committee |
| DMEC | Data Management and Ethics Committee |
| eCRF | Electronic Case Report Form |
| EDC | Electronic Data Capture Form |
| EXSW | Female veterans |
| GAD | Generalised Anxiety Disorder |
| GCP | Good Clinical Practice |
| GDPR | General Data Protection Regulation |
| IM | Iona Mind |
| Iona SHAM CONTROL | Iona Mind SHAM control app |
| IonaFFV | Iona Mind app for Female Forces Veterans |
| ISRCTN | International Standard Randomised Controlled Trials Number |
| ITT | Intention to Treat |
| LICBT | Low-Intensity Cognitive Behaviour Therapy |
| mCBT | Mobile Cognitive Behavioural Therapy |
| MAUQ | Mobile Acceptability and Usability Questionnaire |
| PHQ-9 | Patient Health Questionnaire-9 |
| PI | Principal Investigator |
| pRCT | Feasibility Randomised Controlled Trial |
| PROM | Participant Reported Outcome Measures |
| REC | Research Ethics Committee |
| SAE | Serious Adverse Event |
| SAP | Statistical Analysis Plan |
| SAR | Serious Adverse Reaction |
| SOP | Standard Operating Procedure |
| TIDieR | Template for Intervention Description and Replication Checklist |
| UNEXE | University of Exeter |
| WASAS | Work and Social Adjustment Scale |

Figure 1: Illustration of Feasibility RCT comparing Iona Mind (IonaFFV) AI driven mobile phone app for the treatment of depression in female veterans with a SHAM control app (Iona SHAM CONTROL)


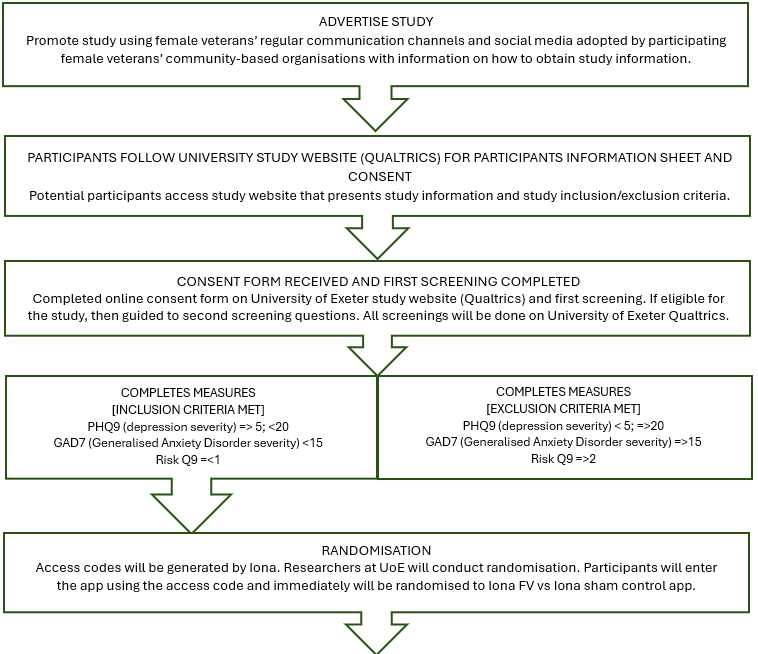


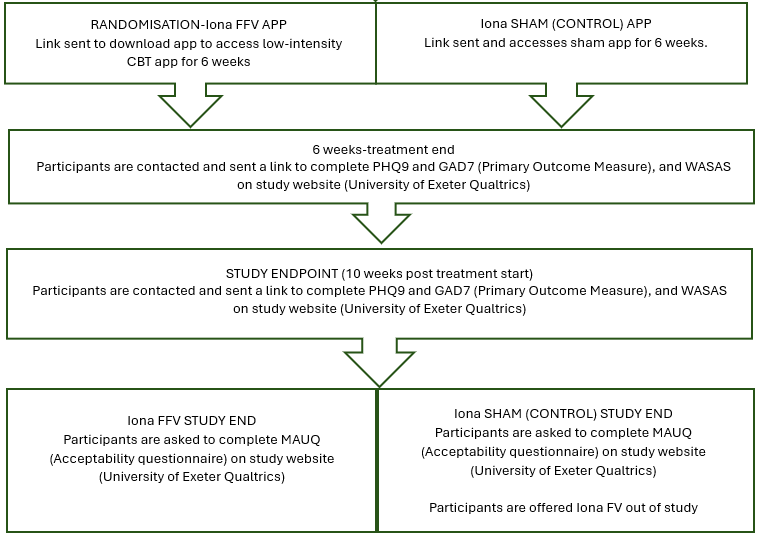


### 1 BACKGROUND

Help-seeking barriers to mental health services experienced by female veterans result in them being underserved and underrepresented compared to female serving, civilian or male Armed Forces Veterans (Leung et al., 2020). Current NHS England data demonstrates 1,500 female veterans only accessing NHS Talking Therapies for Anxiety and Depression or Op COURAGE statutory mental health services per annum. If these numbers are compared with access rates of serving women, the general population, or male veterans they are approximately 50% of what would be expected. Low uptake of mental health services by female veterans in the UK suggests services and interventions are not well adapted to their needs and requires adaptation to enhance engagement.

Research highlights adaptations to areas such as language, imagery and tailoring specific Cognitive Behavioural Therapy (CBT) written self-help interventions to target armed forces veterans enhances acceptability (Farrand et al., 2019). In this study however, participants were exclusively male and the extent that preferences are consistent with those held by female veterans is largely unknown. Furthermore, little is known regarding the extent that preferences may equally be applied to intervention formats such as digital mobile phone apps and if provided through community organisations representing the interests of female veterans. Increased knowledge is therefore important given the small number and wide geographical distribution of female veterans that makes it difficult to offer bespoke and cost-effective sustainable services. Challenges are amplified when current commissioning arrangements remain focussed on statutory mental health services rather than considering wider community-based options. Limited options are significantly problematic given serving females experience high levels of mental health difficulties (Jones et al., 2020), making increased prevalence of mental health difficulties experienced by female veterans likely.

Developing acceptable, scalable and cost-effective interventions delivered by mobile digital apps represents a solution, with good levels of acceptability for female veterans (MeTVeT study; Parkes et al., 2022) justify this proposal to co-develop an app targeting female veterans. Utilising apps to treat mental health difficulties is novel, innovative and can limit stigma, concerns regarding confidentiality or future employment prospects and consistent with female veterans’ self-management preferences (Farrand et al., 2019). When examining the development of apps to support delivery of mental health interventions however, consideration is still required to the evidence-base. Currently, the National Institute for Health and Care Excellence (NICE) restricts recommendations to CBT self-help interventions only when supported by a psychological practitioner for depression (NG222; 2022) and generalised anxiety (CG113; 2020). However, the NHS has begun to successfully implement digital interventions for common conditions such as insomnia via the Sleepio app (Luik et al., 2016) with support provided by AI and is considering increased service provision through non-statutory organisations.

Working with a group of female veterans and others with relevant expertise, this proposal has adapted Ione XSW for this specific group. Following adaptation, the aim of this proposal is to undertake a Phase II Feasibility RCT to explore potential effectiveness, engagement and wider methodological factors of IonaFFV compared to a SHAM control app (Iona SHAM CONTROL). If data suggests IonaFFV has potential to be effective and is acceptable, the study may be used to inform a subsequent Phase III Definitive RCT to treat depression in female veterans. If the app demonstrates acceptability, is engaging and shows promise to be effective, it offers a scalable way to overcome treatment barriers for all female veterans that have currently been underserved.

### 2 RESEARCH QUESTION

**Principal research question:** What are the methodological uncertainties and feasibility challenges in conducting a definitive RCT of the adapted IonaFFV app for female veterans?

**Secondary research question:** Is there potential for the adapted IonaFFV for female veterans to be effective for the treatment of depression and anxiety, is it engaging and acceptable?

### 3 STUDY OBJECTIVE

**Principal objective:** The principal research objective of the Phase II feasibility RCT is to understand methodological uncertainties (e.g., recruitment) associated with running a definitive RCT and to understand intervention acceptability and engagement.

**Secondary objective**: The secondary research objective of the Phase II feasibility RCT is to understand whether there is a potential for the adapted Iona app for female veterans to be effective for the treatment of depression and anxiety.

### 4 KEY WORDS

mCBT, low-intensity, Armed Forces, female veterans, adaptation, AI, app, feasibility, RCT

### 5 STUDY SUMMARY

| **Title** | Feasibility evaluation of a smartphone based Cognitive Behavioural Therapy (CBT) intervention for the treatment of low mood or support for worry management in female armed forces veterans in England, Wales, and Scotland. | |
| --- | --- | --- |
| **Internal ref. no. (or short title)** | Iona female veterans | |
| **Clinical Phase** | II | |
| **Trial Design** | Feasibility RCT with acceptability and usability questionnaire  Parallel group design with randomisation conducted using block randomisation (blocks of 3) and the trial is conducted with an exploratory framework. | |
| **Trial Participants** | Female forces veterans | |
| **Planned Sample Size** | 60 (30 participants per group) | |
| **Treatment duration** | 6 weeks | |
| **Follow up duration** | End of 6-week intervention engagement period and 4 week follow up (10 weeks post randomisation) | |
| **Planned Trial Period** | 10 weeks post-randomisation | |
|  | **RCTs & measures** | **Outcome domains** |
| **Primary/Feasibility/Methodological Outcomes** | Consented, recruited, completed screening, recruitment method, measure completion (baseline, 6 weeks, 10 weeks), drop out, drop out reason | Feasibility |
| **Secondary Outcomes** | PHQ-9/GAD7  Work and Social Adjustment Scale (WSAS) | Depression/Anxiety dependent on lower score on outcome measure at baseline determining severity.  Functioning |
| **Acceptability** | Mobile Acceptability and Usability Questionnaire (MAUQ) | Acceptability and Usability |
| **Intervention** | Intervention: IonaFFV for depression engaged with for 6 weeks intervention engagement period  Control: Iona SHAM CONTROL app developed to match engagement time | |
| **Route of Administration** | Iona app (IonaFFV) for the treatment of depression or Generalised Anxiety Disorder | |

**Table 7** **Study summary**

**NOTE: Secondary outcome measures and acceptability questionnaire will be collected by researchers at the University of Exeter Qualtrics platform.**

### 6 TRIAL MANAGEMENT/RESPONSIBILITIES

A dedicated Lead Researcher who holds a PhD in Social Psychology with 6 years of experience in applied mental health field from the University of Exeter (UNIEXE) and a dedicated Graduate Research Assistant (GRA) will assist with the day-to-day management of the project and be responsible for effective communication and monitoring progress. The wider Study Management Committee will have oversight of the Feasibility RCT through bi-monthly meetings via teleconference or videoconference with the PI to review progress and set targets or directly if approached by the PI or researcher to address a specific question. The trial will be registered with https://www.isrctn.com/ and assigned an ISRCTN number. Researchers have been trained in Good Clinical Practice. We will comply with the UK Policy Framework for Health and Social Care Research and UK Medical Device Regulations. The trial will be conducted to protect the human rights and dignity of participants as reflected in the 2024 version of the Declaration of Helsinki. Trial documents will be retained for a period of 10 years after the completion of the study as detailed in the Patient Information Sheet. Ethical approval will be sought by the UNIEXE Psychology Ethics committee before starting the study.

### 6.1 Participant registration/randomisation procedure

The consent and study screening process will take place on the University of Exeter password protected online Qualtrics system. Female veterans wishing to consider participation will either click on the link or scan the QR code on study advertisement to access the Participant Information Sheet. Contact information for the researcher will be provided on the Participants Information Sheet to enable potential participants to gather more information if requested. Upon receipt of a completed Consent Form, the potential participant is able to access and complete the first screening measures to determine study eligibility. Eligible participants will then be guided to complete the second screening measures to determine study suitability based on participants symptoms of anxiety and depression and well-being – PHQ9 and GAD7. Second screening will also serve as baseline measures for participants. Where inclusion criteria are met, and upon completion of the sociodemographic questions, the participant will be randomised in blocks of 3 into either of the study arms and automatically registered on to the trial. Once randomised, participants will be sent a code to download the app.

### 6.2 Data management

Only authorised UNIEXE research personnel will have access to the password protected Qualtrics database. No unauthorised access will be possible. A separate list linking codes with mobile phone number and email addresses will be kept in a secure place and only accessed by Lead Researcher. Only aggregated data will be shared with UNIEXE research team. The data will be introduced and analysed by computers. As for Internet use and monitoring by means of mobile apps, data protection systems will be designed (using secure passwords, encryption, etc.). The database is only accessible via a password. Also, to protect all information, we will follow the AES (Advanced Encryption Standard) strategies for personal password use and data encryption. The study researchers will not reveal data from which personal and health information about the participants could be deduced. The same principles will be taken into consideration in the dissemination of data in the publication of scientific papers and the presentation of research reports at scientific conferences.

6.2.1 Database infrastructure

The project will use a distributed electronic database (managed by UNEXE) during the project that will store all the downloaded cohort data and clinical trial data. Within the clinical trials, UNEXE will be in charge of the set-up and management of the database. The equivalent of anonymised electronic Case Report Form (eCRF) data will be set-up and entered in a Qualtrics, which is fully validated. The eCRF and associated database will be automatically populated from the responses entered by participants via websites and app platform: data will be encrypted and pseudonymised immediately after downloading from the website or app and then stored securely and converted into an electronic database suitable for analysis. Lead Researcher will code-link personal information to the rest of the data. This coding spreadsheet will be password protected and only accessed by the lead researcher. The rest of the research team will only have access to the aggregated data. Data will be routinely backed-up during and after the project to ensure the availability of all the information.

### 6.2.2 Data Management Plan

The Data Management Plan will describe how the data will be exploited, checked, shared, curated and preserved. Thus, the procedure for granting access will be detailed and the mechanisms to access the data after the project will be described. It should be noted that no data will be collected or used without the explicit informed consent of the participants.

### 6.2.3 Data format and types

Standard data formats will be used during the project and will be compliant with Clinical Data Interchange Standards Consortium – Clinical Data Acquisition Standards Harmonisation (CDISC-CDASH) standard. Data types will include Volunteer data: Demographics and information provided by participants on the study questionnaires.

### 6.2.4 Data exploitation

All information will have a digital format that will be handled in accordance with European and national data protection regulations. A mechanism to request access, mine, exploit, reproduce or disseminate data generated in the framework of this project will be put in place.

### 6.3 Data protection/confidentiality

### 6.3.1. Data protection

The conduct of the project will comply with GDPR. Adequate measures to ensure data protection and confidentiality will be duly taken into account by the research team. Local and national rules on data protection will be followed, and no personal information of participants will be transferred unless such transfer is essential for the conduct of the trial.

All research data will be stored on a UNIEXE database linked to the unique identifier ID to pseudonymise all information collected. Before taking part, all participants are provided with Participant Information Sheet which also includes information on privacy policy. At initial screening potential participants are not asked to provide their email addresses contact details (i.e., mobile phone number). If participants are eligible to take part after initial screening, they are asked to complete the second screening, which also serves as the baseline measure, sociodemographic questionnaire and provide their email address and mobile phone number. Phone numbers are collected to send reminders to participants. The database contains all the baseline (i.e., second screening) and follow up data collected from the EDC system. The codes linking contact information with the database containing outcomes will be destroyed as early as is legally required (no earlier than all data being archived – 3 months post study endpoint) – data (including participant details and consent) may need to be retained and not deleted for a longer period due to future research indicators that may require researchers to contact the individual or actions taken by participant against the research. This approach has proven successful in prior digital interventions for wellbeing and has been approved by multiple institutional and National Health Service research ethics in the UK; adaptations will be made as necessary for specific local ethical requirements.

Only the UNIEXE Lead Researcher will have access to the database that connects the ID number to a person, their email address and mobile phone number which will only be used to send reminders to participants regarding assessments. Email reminders will never contain personal or other information about the collected data, but remind participants, in a general manner, about open tasks. Only the UNIEXE study researcher MJ will be set up with a username and password to access the database that stores the personal data and hold allocation details to ensure blinding is maintained for all other members of the research team. The database containing pseudonymised data is only accessed by UNIEXE research team.

Participants can request a copy of the study report by ticking a box on the consent form. The email addresses of those who express interest will be stored separately from other data on a secure, password-protected University of Exeter server, accessible only to the Lead Researcher. Once the final report is distributed, these email addresses will be permanently deleted.

### 6.3.2 Digital Information

Files containing digital information will be encrypted with password-protection where appropriate and stored on the UNIEXE secure network (not a local ‘C’) drive. Where local copies are required for processing or transfer preparation, it will be ensured that the target workstation is compliant with all UNIEXE security policies and that they are followed. This is particularly important for laptops/netbooks/portable workstations, especially about encryption and will be confirmed by UNIEXE before transferring data. The relevant university guidelines and policies will be followed (e.g., the UNIEXE Information Governance (https://www.exeter.ac.uk/departments/cgr/ig/policy/), Security Policy (https://www.exeter.ac.uk/departments/cgr/ig/informationsecurity/), Computing Regulations (https://www.exeter.ac.uk/staff/policies/calendar/part1/otherregs/its/) and University guidelines for portable and removable devices (https://www.exeter.ac.uk/media/universityofexeter/it/recordsmanagementservice/policydocuments/Information_Security_for_Portable_and_Removable_Media_Devices_Policy_4.0.pdf). Participants identifiable data will not be stored on home computers, personal laptops, unencrypted memory sticks, CDs, handheld devices, digital cameras or other imaging equipment even if they are password protected. An encrypted memory stick may be used if required.

All data generated will be stored by UNIEXE in encrypted and password-locked files behind a secured firewall operating within a university environment with state-of-the-art safety protection measures, and transmission of information via electronic means will be performed using encrypted data files. The exact process for data storage and encryption for the data processors are outlined in the data management plan.

### 6.3.3. Participant data

Participant confidentiality and welfare will always be maintained as the highest priority. All research staff with access to data is subject to professional secrecy during and after the trial. During the course of the project, we do not anticipate any sharing of individual participant data from this clinical trial outside of the UNIEXE.

Anonymised data (including Armed Forces served in and date of leaving, socio-demographic information, platform usage information) will not be deleted until the completion of the analysis of the data plus the mandatory period for retaining research data (at least ten years in the UK).

The database containing link codes, which connect participants to their study data, will be retained for 3 months after the study's completion. This period ensures participants can exercise their right to withdraw their data and addresses any potential actions taken by participants concerning the research. After this period, this database will be securely and permanently deleted.

###

### 6.3.4 Responsibility

The UNIEXE as sponsor of the trial is the data controller. The controller has the responsibility to ensure that the security and access arrangements for the database comply with the Data Protection Act (2018), and that all data processing and locally held personal data are registered with the host institution according to their employer’s processes. Because this trial involves the processing of personal information the Information Commissioner’s Office (ICO), will be notified accordingly.

Legal data transfer agreements will be written and signed between the data controller and data processors prior to any participant being recruited, where appropriate. These agreements will be confirmation that the data processors will adhere to GDPR regulations, which protect and safely store participant personal and outcome data.

A common data protection and privacy policy authorised by the sponsor, and the UNIEXE data protection team will be available on the study website. The screening website will also email this policy to consenting participants with the information sheet and consent form or provide it as a download.

### 6.3.5 Data monitoring

Data will be accessed by the researchers on a regular basis (typically at least weekly) to check recruitment numbers and data quality and to monitor that all processes are working correctly. Detailed checks will occur early in the project to confirm that all systems are working properly. To download data the Lead Researcher has to login via username and password.

### 6.3.5a Data monitoring committee

An initial meeting held with the DMC determined the role of the DMC and frequency of reporting throughout recruitment and study follow up. The Chair of the DMC (Professor Andy Bacon) will be updated weekly as to study progress and informed regarding adverse events. He will organise meetings to discuss study progress with wider members of the DMC comprising a female armed forces officer, NHS armed forces veterans service lead, and regional commissioner. The DMC is independent of the sponsor and funder. At the end of the study, prior to the randomisation code being broken, a written draft results section will be presented to the DMC alongside the aggregated data to ensure compatibility.

### 6.3.6 Breach of confidentiality

Occasionally records containing personal data that should not have been disclosed, (e.g. an e-mail with a data file containing identifiable details) may be received by a staff member from an internal or external source. In such situations, the member of staff should contact the person who sent the data and make them aware of the breach of confidentiality. The records received should be either promptly deleted or any identifying details thoroughly erased. All suspected breaches would be investigated, documented in the study file and reported to the Sponsor as appropriate, following an established data breach the UNEXE procedure will be followed.

### 6.4 Trial documentation and archiving

The materials for the trial will be submitted with this study protocol.

Source documents and trial-related electronic and other data. Stored safely and in accordance with the requirements of the GDPR and UK Data Protection Act (2018), no longer than legally required (for a minimum of ten years) or as stipulated by the Sponsor’s requirements.

Data Access: Post-analysis, the final anonymised dataset will preferentially be stored in Open Research Exeter (ORE), the UNIEXE open access repository.

Interoperability: Source data will be stored in Microsoft SQL server, formatted to maximise fidelity. This can be transposed and converted during the analysis stage into any format required. For the Open Research Exeter repository XML or CSV with a separate data dictionary is recommended.

Archiving: Items submitted to ORE will be retained indefinitely. ORE content is securely held on UNIEXE servers and regularly backed up according to current best practice. The ORE team will also ensure continued readability and accessibility of content, including the migration to new file formats where necessary.

### 6.5 Authorisation of Participating Site

The study is online and solely located at the UNIEXE who will act as sponsor to the research programme and authorise after ethical approval has been granted. The Chief Investigator and Lead Researcher are familiar with applicable Standard Operating Procedures, risk assessment procedures, and agree that Good Clinical Practice (GCP) and General Data Protection Regulation (GDPR) standards will be followed.

### 7. PARTICIPANT INCLUSION/EXCLUSION CRITERIA

**Table 8 Inclusion/Exclusion criteria**

| **Inclusion** | **Exclusion** |
| --- | --- |
| **Initial screening** | |
| Aged 18 or over | AGED <18 |
| Ex-servicewoman | Not ex-servicewoman |
| Resident in Great Britain | Not resident in Great Britain |
| Ability to read and understand English | Unable to read and understand English |
| No history of psychosis, mania, substance/alcohol dependence | History of psychosis, mania, substance/alcohol dependence |
| Ability to download app | Not able to download app |
| Access to smartphone with internet access | No access to smartphone or access internet |
| Not changed or started ADM in last month | Changed or started ADM in last month |
| Not currently receiving mental health support | Currently receiving mental health support |
| **Second screening/Baseline** | |
| PHQ-9=>5; <20 | PHQ-9<5; =>20 |
| GAD7<15 | GAD7=>15 |
| Suicide risk: PHQ-9 Q9=<1 | Suicide risk: PHQ-9 Q9=>2 |

### 8. TRIAL PROCEDURES

### 8.1 Participant identification

We will recruit from participating Armed Forces Veteran specific community and charitable organisations, social media and through opportunistic sampling. Posters, emails, websites, presentations, and social media will all advertise the study to female veterans who wish to tackle anxiety and depression. Recruitment of participants will be through following routes commonly adopted and used by the community/charitable groups supporting the study such as:

• Local promotion and email circulars commonly adopted by the community/charitable organisations and local promotion (e.g. posters, emails, newsletters, signposting by staff).

• Multiple traditional and social media (posting and advertising on Facebook, LinkedIn, Instagram, SnapChat, Google, MySpace, X/Twitter, TikTok, through media influencers/vloggers, etc adjusted by cost/frequency as needed); social media analytics will be used to enhance recruitment.

• Snowballing approaches.

### 8.2 Screening

Participants will be screened online - those under 18, not an EXSW, not resident in the UK, history of psychosis, mania, substance/alcohol dependence, no access to smartphone or internet, changed or started ADM in the last month, and currently receiving mental health support will automatically be screened out during the first screening. Website users who report having current risk, based on PHQ9 and GAD 7 during the second screening will be taken to the feedback screen where they will be advised we are sorry to hear that they are feeling that way, their safety is of our utmost concern, to please contact their GP or other Armed Forces related risk management organisation and to give them sources of online help and support. Contact details for appropriate Armed Forces specific organisations to seek further help and support risk management have been informed by the ex-Armed Forces Veterans Lead for the NHS and co-investigator on the research.

### 8.3 Consent

Potential participants will be initially routed to, or seek out, the UNIEXE Qualtrics page and will be provided with the Participant Information Sheet. If interested in participating, they will select a checkbox that takes them to the Consent Form and data protection policy to review on the trial electronic platform. Study eligibility and inclusion criteria will be clearly stated, and participants asked to electronically sign the Consent Form and date. The Consent Form asks for consent to undertake the initial and secondary screening, taking part in the trial as well as confirmation that the participant understands the nature of the study and meets inclusion criteria. Participants are asked to confirm that they meet each criterion. If eligible, participants will be asked to provide an e-mail address and mobile phone number.

If a participant completes the Consent Form and is eligible for the trial, they will be given an e-mailed confirmation that they are in the study, a unique study participant trial number and instructions indicating how, as per randomisation scheme, to download IonaFFV or Iona SHAM CONTROL on either an Android or Mac (IOS) based mobile phone. They are also provided with contact details (email, and/or telephone number as available) for the Lead Researcher if further study information is wanted or they are struggling to download IonaFFV or Iona SHAM CONTROL.

### 8.4 Randomisation scheme

The feasibility RCT will follow MRC Complex Interventions Guidelines and relevant CONSORT reporting requirements with a pre-registered trial protocol. Randomisation will be in blocks of 3 with randomisation to the 2 arms (IonaFFV vs Iona SHAM CONTROL) conducted automatically by Qualtrics survey platform to minimise selection bias and maintain methodological rigor. Following randomisation IonaFFV or Iona SHAM CONTROL will be automatically sent a link to download the app.

### 8.5 Protection from bias

We will adopt prior ISRCTN registration and seek publication of the trial protocol. Randomisation will be conducted by researchers at the University of Exeter using codes generated by Iona, allocation sequence of participant allocation and of allocation sequence and prevent selection bias and confounding. We will use standardised self-report outcome measures with study data collected automatically on the UoE Qualtrics system. The use of self-administered measures will eliminate observer bias. A detailed statistical analysis plan will be prepared before any analysis is conducted. The trial will be double blinded so the trial participants and data collectors and analysts will remain blinded to group allocation. Prior to the randomisation code being broken by the research team the statistical analysis will be conducted and Results section written with reference to group A and group B and shared with the project steering group and Data Monitoring and Ethics Committee to ensure they are happy the written Results section matches the data analysis to ensure they are satisfied they are representative of each other. Attrition bias will be minimised by having robust trial procedures to prevent data loss such as email and text to encourage follow ups.

### 8.6 Method of implementing the randomisation/allocation sequence

Participants will be randomised by pseudorandom number generation. Eligible participants will receive an email confirmation which provides a download link and unique participant code to each participant accepted into the trial, and they will be prompted to download the app as soon as possible. Once selected according to the randomisation sequence either IonaFFV or Iona SHAM CONTROL, participants will download the app on their phone. Participants are blinded to the randomisation.

Should a participant fail to download the app and enter the code, an automated sequence of emails at the rate of 1 per day over a 7-day period will be sent to the participant reminding them to do so and reiterating the download and sign-up instructions. After this time no further contact with the participant will be made.

Detailed procedure for randomisation:

1. Participants are randomised in blocks of 3 (30 participants per arm) into the 2 arms (IonaFFV versus Iona SHAM CONTROL) on an intention-to-treat basis.
2. Block randomisation will continue until 60 (30 each arm) are randomised. As eligible participants complete the initial screening and demographics questionnaire they will be randomised in blocks of 3 to either the control or intervention arm by the Postdoctoral Research Associate or the Graduate Research Assistant.

3.       The system will record the allocation and the date randomised.

### 8.7 Blinding

All follow-up research data will be routinely collected by the UNIEXE Qualtrics system, with the system sending reminders by email and text message. The Lead Researcher will undertake the initial analysis which will be discussed with the wider research team and agreed with the written analysis section by the Trial Steering Committee. Participants and data analysts will be blinded.

Philip Howson (Iona Mind) will provide a set of download codes for group A and group B. The researchers will not know which group of codes contains the access to the intervention or control arm. The researchers will randomly allocate these codes to eligible participants in blocks of 3.

Accidental unblinding may occur if one of the research team receives an email from a participant with questions or comments containing identifiable information about the treatment arm they are in. If this happens, the researcher will notify the research team and will not conduct any analysis to allow the blinded researchers to conduct the analysis.

### 8.8 Baseline data

Baseline data will be collected after participants sign the consent form but before randomisation. Here, data from second screening, if participants meet the study inclusion criteria, serves as baseline data. This is when the participant completes the PHQ9 and GAD7 on University of Exeter Qualtrics for the first time.

### 8.9 Trial assessments

Assessment will take place at baseline and then at 6 weeks post treatment start (equating to end of treatment) and 10-weeks post baseline representing the study endpoint. At both of these timepoints the participant will receive a request to complete the outcome measures with a URL included to take them to the UNIEXE Qualtrics page. The results of the assessments are only to collect research data for the trial and will not be provided to anyone outside of the study. The only exception to that would be if a participant indicates suicide risk and asks us to provide their assessment results to their medical practitioner. They would need to give written consent for this and provide us with the contact details to do so.

At the end of treatment, all participants will be asked to complete the MAUQ

to determine the acceptability and usability of the app.

**Table 9 Assessment Schedule**

| **Procedures** | **Initial screening** | **Baseline (second screening)** | **6 weeks post initial app engagement** | **4 weeks follow-up**  **(10 weeks post initial app engagement)** |
| --- | --- | --- | --- | --- |
| Informed consent | Yes | Yes | No | Yes |
| Demographics | Yes | Yes | No | No |
| Mental Health History | Yes | No | No | No |
| Eligibility assessment | Yes | Yes | No | No |
| Randomisation | No | Yes | No | No |
| Access to intervention | No | No | Yes | No |
| Assessment of wellbeing and depression | No | Yes | Yes | Yes |
| Assessment of current functioning | No | Yes | Yes | Yes |
| Assessment of usability and engagement | No | No | No | Yes |
| Feasibility outcomes | No | No | No | Yes |
| Adverse event assessments | No | No | Yes | Yes |

### 8.10 Long term follow-up assessments

Participants will be followed up at 6 (Treatment end) and 10 weeks post baseline (4 weeks follow up) post treatment end.

### 8.11 Payment

Participants will be paid £30.00 in electronic vouchers for completing the measures after completion of the 4 weeks follow up (10 weeks). Participants indicating, they wish to withdraw from the study will receive this payment.

- £20 for those who complete the 6-week questionnaire (but not follow up),

- £10 for those who drop out following downloading the app.

### 8.12 Withdrawal criteria

At any time, participants can choose whether they want to stop using the IonaFFV or Iona SHAM CONTROL intervention, or if they want to withdraw from the trial completely (including all assessments). A log will be kept of the participant number and date of all withdrawals from the trial. Participants who met the inclusion criteria at baseline will not be replaced. Participants that did not meet the criteria at baseline can be replaced and their data removed from the data set. Participants who withdraw from the trial will not be followed up. Once a participant withdraws from the study, they will still be able to use the app until the end of the study period if they choose.

### 8.13 End of trial

The ethics committee and Sponsor will be notified at the end of the trial or within 15 days if terminated early.

### 8.14 Assessment of engagement at 6-week treatment end

Participants in the IonaFFV arm will be asked to complete the MAUQ at treatment end.

### 9 INTERVENTIONS

### 9.1 Iona mobile phone app adapted for Female veterans (IonaFFV)

The intervention is reported according to the Template for Intervention Description and Replication (TIDieR) Checklist (Hoffman et al., 2014).

**Table 10 TIDieR Checklist**

| Item | Description |
| --- | --- |
| Brief Name | Iona: Female forces veterans (IonaFFV) |
| Why | Help-seeking barriers to mental health services experienced by female veterans result in them being underserved and underrepresented compared to male veterans, male /female servicing personnel or civilians (Leung et al., 2020). When compared to access rates of serving women or male veterans, they are approximately 50% of what would be expected. This is problematic given serving females experience high levels of in-service adversity making increased prevalence of mental health difficulties experienced by female veterans likely. Developing acceptable, scalable, and cost-effective interventions delivered by mobile digital apps targeting female veterans presents a potential solution to improving access. However, efforts to ensure interventions are adapted to meet the preferences and needs of female veterans to enhance acceptability and engagement are first required. Using a focus group methodology and working alongside a female veterans involvement group, an understanding of adaptations needed to enhance acceptability have now been used to adapt the XSW app for female veterans. However, whilst enhancing acceptability, little is known regarding the potential effectiveness of the app in the treatment of depression or Generalised Anxiety Disorder. The need to undertake a feasibility RCT to examine potential effectiveness of the app versus a SHAM control app is therefore needed before considering wider evaluation and potential implementation. |
| What | Mobile phone app for the treatment of MDD informed by the clinical protocols commonly adopted for the treatment of depression or Generalised Anxiety Disorder by face-to-face CBT. During initial engagement with IonaFFV participants will be informed regarding the best CBT technique based on outcome measures taken at baseline. Following participants gaining an understanding of their depression or GAD using a CBT model they are provided with an option of evidence-based CBT techniques for depression. Options include Behavioural Activation, Cognitive Restructuring with Behavioural Experiments and Worry Management (Problem Solving with Worry Time). Support using any of the intervention techniques is provided by AI for the duration of the 6-week treatment period. |
| Who Provided | The intervention is solely provided in-app, there is no human support. |
| How | Participants will be encouraged to use the app to engage with the CBT technique for 6 weeks. Support is provided in-app by an AI driven chatbot, no human support is available. |
| Where | The intervention is provided by the IonaFFV app, employing the CBT techniques with support provided in-app by an AI driven chatbot. |
| When and How Much | Following download of the app, participants will be encouraged to engage with the intervention on their own over the next 6 weeks. It will be emphasised within the app that engaging for a manageable amount of time on a regular basis is better than trying to use the intervention for a long period of time periodically. However, the exact amount of time the participant engages with the intervention will be determined by them. |
| Tailoring | The IonaFFV intervention has been extensively adapted and co-produced for es-servicewomen through focus groups and wider consultation prior to this Feasibility RCT. |
| Modifications | The intervention used within the study is an adaptation of the Iona app for the general population developed by Iona Mind Inc. Modifications to the IonaFFV app for female veterans are largely focussed on language, examples, armed forces related quotes, imagery. |
| How Well | Log-data regarding engagement with the app and specific lessons and features with time engaged will be taken for each participant. Furthermore, the MAUQ will take participant responses regarding general usability. |

###

### 9.2 SHAM control app

Participants randomised into Iona SHAM CONTROL app consisting of approximately the same number of lessons as Iona FV, with repeatable “meditation-like” exercises. The “meditation-like” lessons will consist of text, video and audio, similar to that of the IonaFFV app, and the exercises will again be text, video and audio, with the length of each lesson and exercise adapted to be approximately the same as the average engagement time per session with IonaFFV in order to match time on task for the user. Iona SHAM CONTROL will be delivered within the same app framework using approximately the same app and cloud infrastructure as IonaFFV to ensure a consistent look and feel and user experience across both apps. Similar to IonaFFV, notifications and emails will encourage the user to engage daily with the techniques with the aim of achieving a similar usage pattern between both apps. Iona SHAM CONTROL also contains modules on therapies that while commonly recognized are not evidence-based, such as Dream Analysis, again delivered using text, images and audio in a similar manner to IonaFFV. Such modules will be accompanied with related tooling such as a dream diary, presented similarly to the activity diary in IonaFFV, again with the aim of matching time on task and approximate user-experience to IonaFFV without containing any active evidence-based treatment.

### 10 OUTCOME MEASURES/ENDPOINT

### 10.1 Demographic Data

Demographic data will be taken at baseline and address:

- Age
- Gender identity (M/F/neither/both)
- Sexual orientation
- Race/ethnicity
- Service
- Length of service
- Time left armed forces
- Rank leaving service
- Early Service Leaver
  - Optional free response box available to provide reason

### 10.2 Outcome measures

**Table 11 Outcome measures**

| **Measure** | **Description** | **Reliability and Validity** |
| --- | --- | --- |
| **Primary/Feasibility/Methodological Outcomes** | | |
| **Feasibility** | To collect data on number of participants consented, recruited, completed screening, and to assess recruitment method, measure completion (baseline, 6 weeks, 10 weeks), drop out, drop out reason | NA |
| **Secondary Outcomes** | | |
| **Patient Health Questionnaire-9** (**PHQ-9**; Kroenke et al., 2001) | 9-item participant rated questionnaire assessing frequency of symptoms of depression over the last 2 weeks. 4-point scale for each item, with anchors at 0=not at all, 1 = several days, 2= more than half the days, 3 = nearly every day. Unidimensional scale. Available in English, Spanish, German, and Dutch versions. | Leading measure of depression widely used in clinical trials, clinical practice, and as part of the NHS Quality and Outcomes Framework (QOF) for UK primary care and Improving Access to Psychological Treatments (IAPT) Minimum Data Set (MDS). Cronbach’s α =0.89 in primary care, test-retest reliability (ICC) 0.84 after 48 hours. Validation studies indicate positive correlations with measures of functional status (r=0.73), disability days (r=0.39), and symptom-related difficulty (r=0.55) At cut-off of ≥ 10, excellent specificity (0.88) and sensitivity (0.88) with diagnoses of major depression by structured interview, replicated in a UK population (sensitivity 0.80; specificity 0.92). |
| **Generalised Anxiety Disorder-7 (GAD-7**; Spitzer et al., 2006) | 7-item participant rated questionnaire assesses frequency of symptoms of anxiety over the last 2 weeks. 4-point scale for each item, with anchors at 0=not at all, 1 = several days, 2= more than half the days, 3 =nearly every day. Unidimensional scale. Available in English, Spanish, German, and Dutch versions. | Leading measure of anxiety widely used in clinical trials, clinical practice, and as part of the UK NHS IAPT MDS. Cronbach’s α =0.92, test-retest reliability ICC = 0.83. Convergent validity good, r =.72 with Beck Anxiety Inventory, r = 0.74 with Symptom Checklist-90 anxiety scale. |
| **Work and Social Adjustment Scale (WSAS**; Mundt et al., 2002) | 5-item participant rated questionnaire assesses impaired functioning, rated from 0 not at all impaired to 8 severely impaired, with respect to work/education, home management, social leisure, private leisure, and close relationships. Unidimensional scale. | Leading measure of functioning, widely used in clinical practice, and as part of the UK NHS IAPT MDS. Cronbach’s α range from 0.70 to 0.92, test-retest reliability ICC = 0.73. Interactive voice response administration correlated 0.81 to 0.86 with clinician interviews. |
| **Acceptability** | | |
| **mHealth App Usability Questionnaire (MAUQ**; Zhou et al., 2019). | 21-item participant rated questionnaire measured on a 7-point scale ranging from strongly disagree (1) to strongly agree (7). The questionnaire measures acceptability and usability for interactive mental health apps. Three domains explore Ease of Use and Satisfaction, Usefulness and System Information Arrangement. Unidimensional scale. Given IonaFFVis not intended to support communication with healthcare providers from within the app, 5 questions were removed resulting in a 15 item measure. This has been undertaken in other research focused on an Armed Forces community (Parkes et al., 2023), where the app did not support communication with other health care providers. | Commonly adopted measure to rate mobile phone apps across several dimensions. Scale has good internal consistency with Cronbach alpha values of 0.895, 0.900 and 0.829 respectively. |

### 10.3 Timeframe for administration of demographic and outcome measures

**Table 12 Outcome measures timeframe**

|  | **Time Period** | | |
| --- | --- | --- | --- |
| **Outcome Measure** | **Baseline (second screening)** | **6 Weeks Post Treatment Start (Treatment End)** | **10 Week Post Treatment Start (Study End Point)** |
| **Demographic** | **X** |  |  |
| **Primary Outcome (Either Dependent on Second screening/Baseline Severity - Other Secondary)** | | | |
| **PHQ-9** | **X** | **X** | **X** |
| **GAD-7** | **X** | **X** | **X** |
| **Secondary Outcomes** | | | |
| **WSAS** | **X** | **X** | **X** |
| **MAUQ** |  |  | **X** |
| **Feasibility Outcomes** |  |  | **X** |

### 10.4 Primary endpoint/outcome

The primary endpoint of the trial is at 6 weeks post treatment start (treatment end).

### 10.5 Secondary endpoints

The secondary endpoint is at 10 weeks post baseline (4 weeks post treatment end follow-up).

### 10.6 Exploratory endpoints

There are no exploratory endpoints.

### 10.7 Feasibility outcomes

**Table 10 Feasibility Outcomes**

| **Study Recruitment** | | | |
| --- | --- | --- | --- |
| Number of people accessed study web site |  | | |
| Number of people complete screening questions |  | | |
| Number of people screened out | If Yes - Reason | Report by demographics | |
| Number of people consented |  | Report by demographics | |
| **Randomisation** | | | |
| Number of people randomised |  | Report by demographics | |
| Participant blinding randomisation maintained | Yes | | No |
| **Outcome Completion Both Study Arms** | | | |
| Number completed baseline outcome measure | Data completion of each item for each outcome measure | | |
| Number completed end of treatment outcome measures (6 weeks post baseline) | Data completion of each item for each outcome measure | | |
| Number completed final outcome measures 10-week post baseline | Data completion of each item for each outcome measure | | |
| **Recruitment Method** | | | |
| Recruitment method adopted | Number recruited | | |

### 11. STATISTICS AND DATA ANALYSIS

Statistics will be undertaken by the Lead Researcher.

### 11.1 Sample size calculation

As a feasibility RCT there is no sample size calculation. Rather, data will be used to inform a sample size calculation for any subsequent definitive RCT if the data demonstrates potential effectiveness.

### 11.2 Planned recruitment rate

Recruitment of 60 participants into the study will take place online over 16 weeks within a single study site (UNIEXE). Recruitment rate within this timeframe represents a feasibility outcome.

### 11.3 Statistical analysis plan

A detailed statistical analysis plan (SAP) is to be produced; the main points of the statistical analysis are summarised here. All participants will be included in each analysis, separated by arm. Missing data will be reported descriptively. No imputation will be performed due to the feasibility nature of the study, but patterns of missingness will be explored to inform future trial design.

### 11.4 Summary of baseline data and flow of patients

The analysis and presentation of the trial will be in accordance with CONSORT guidelines (Schulz et al., 2010). Recruitment, intervention uptake, outcome completion rates and attrition will be reported (with 95% CIs) and shown on a study flow diagram.

Participants may elect to withdraw from the study if they wish to do so at any time and for any reason (including perceived harms or lack of efficacy of intervention). This can be easily done by discontinuing use of the randomised app.

Participants will be withdrawn from the study entirely if discovered as ineligible at the time of recruitment. As a self-help psychological intervention, we do not anticipate significant iatrogenic effects or side-effects requiring individual discontinuation.

### 11.5 Primary analysis

Primary analyses will be conducted on methodological and feasibility data, such as recruitment rate and source, completion of baseline measures, study attrition etc. For outcomes related to study feasibility (see Table 10).

### 11.6 Secondary analyses

Will be undertaken on either the PHQ-9 or GAD-7, the WSAS and MAUQ (see Table 12 for administration timepoints).

### 11.7 Outcome reporting

Continuous outcomes will be reported descriptively (mean and standard deviation (SD). To explore potential effect sizes and confidence intervals, inferential comparisons will be reported between the two conditions (1) IonaFFV; (2) Iona SHAM CONTROL (control).

### 11.8 Interim analysis and criteria for the premature termination of the trial

No interim analyses are planned. If during second screening the participant records >1 PHQ-9 Q9, they will be directed to the SOS screen where details regarding help seeking for risk will be provided, which has been tailored for Armed Forces veterans. They will receive a message indicating that the app is not suitable to meet their needs and encouraged to contact one of the organisations identified. In order to detect potential harm, all occasions where participant score on item 9 of the PHQ-9 has elevated to 2 or 3 will be recorded by the Lead Researcher as an Adverse Event (AE). Elevation will result in classification as an Adverse Event and result in trial discontinuation for the participant.

### 11.9 Other statistical considerations

Primary and secondary analyses will be performed by the Lead Researcher. The results will be discussed and interpreted prior to the unblinding of group allocation.

### 12. ADVERSE EVENTS

### 12.1 Participant welfare and safety

There is no known health risk associated with any of the assessments or either IonaFFV or Iona SHAM CONTROL. The risk concerning participation in this study is believed to be low. Further, we anticipate that IonaFFV will reduce low mood or worry. In our experience from previous projects, participants are happy to participate and enjoy the assessment tasks. We will strive to use tasks that the participants experience as motivational and reinforcing whenever possible. This will also ensure a low attrition rate.

Because the trial is only focussed on people with mild to moderate depression or anxiety, the initial screening process will exclude anyone with a history of severe psychiatric disorder and those reporting elevated suicidality (PHQ-9>1). These individuals will be automatically guided towards appropriate information and sources of help. This process means that individuals likely to have significantly increased risk (e.g., for self-harm and suicidality), and/or for whom more intensive psychological and psychiatric treatment is appropriate, will not be included in the study.

Other than the intervention failing to produce an effect, there is nothing in the literature to suggest possible adverse effects of the assessments and interventions. As with all psychological interventions, individuals reflect on their difficulties, which can produce temporary increases in distress, but no more than would commonly occur in daily life. Versions of techniques within the intervention are commonly adopted within a general adult version of the Iona app previously employed as in an NHS Talking Therapies for anxiety and depression service and with no detected harmful effect. As such, the intervention within the trial may benefit individual participants. The likeliest outcome for users who do not find the intervention of benefit is their disengagement from it. In addition, all participants receive more intensive monitoring, with processes within IonaFFV and Iona SHAM CONTROL to identify and direct all relevant participants to potential sources of help.

### 12.2 Risk management processes

As part of our policy for addressing risk and prioritising the welfare of participants there are 2 main processes in place.

The main indicators of harm will be completion of the PHQ-9 questionnaire by the participants in both study arms at all assessments (baseline, 6 weeks, 10 weeks). The PHQ-9 study questionnaire will be automatically screened by the AI function of the apps for signs of distress (defined as scores above 20 on PHQ-9 for depression or reports >1 on Question 9 of the PHQ-9). Outside of the research there is also a function within the apps to identify aspects of risk and through the SOS button, which if selected will automatically provide participants with recommended advice and signpost towards help (e.g. NHS and Armed Forces specific support services).

We will record all occasions where risk within IonaFFV or Iona SHAM CONTROL app has been highlighted and recorded and reported to the Trial Management Group and Sponsor to determine whether events are related to the treatments and to take appropriate action.

### 12.3 Definitions

Standard definitions for Adverse Events etc are in Table 13 below. Because the current interventions are digital self-help rather than a medicinal product and involve no biological agent, it is not appropriate to define adverse events etc re any untoward medical occurrence – rather as a psychological intervention, appropriate adverse events would include those related to mental state and behaviour. Furthermore, given the study has no involvement from a mental health or community-based service it will not be possible to record Serious Adverse Events/Reactions or Suspected Unexpected Serious Adverse reactions, only Adverse Events (AEs) captured within the app, or Adverse Reactions (ARs) as determined by the Sponsor can be recorded.

1. Adverse Events (AE) may include significant worsening symptoms of anxiety, worsening symptoms of depression, as operationalized by a deterioration of movement from ‘mild or moderate’ (PHQ-9=>10); to ‘severe’ (PHQ-9=>20) levels of symptoms on the PHQ-9 at 6 weeks assessment or from 6 weeks assessment to 10 weeks assessment.
2. Adverse Reactions (AR) same as AE but where a relationship to the app can be determined.

The following definition is adapted to accommodate the status of the intervention as digital self-help (Table 13).

**Table 13 Definitions of Events**

| **Term** | **Definition** |
| --- | --- |
| **Adverse Event (AE)** | Standard: Any untoward medical occurrence in a participant to whom a medicinal product has been administered, including occurrences which are not necessarily caused by or related to that product.  Adapted: Any deterioration in mental state or behaviour in a participant to whom the intervention has been administered, including occurrences which are not necessarily caused by or related to the intervention. |
| **Adverse Reaction (AR)** | Standard: An untoward and unintended response in a participant to an investigational medicinal product which is related to any dose administered to that participant.  The phrase "response to an investigational medicinal product" means that a causal relationship between a trial medication and an AE is at least a reasonable possibility, i.e., the relationship cannot be ruled out.  All cases judged by either the reporting medically qualified professional or the Sponsor as having a reasonable suspected causal relationship to the trial medication qualify as adverse reactions.  Adapted: An untoward and unintended response in a participant to an intervention which is related to any feature of the digital intervention administered to that participant.  The phrase "response to an intervention" means that a causal relationship between an intervention and an AE is at least a reasonable possibility, i.e. the relationship cannot be ruled out.  All cases judged by the Sponsor as having a reasonable suspected causal relationship to the intervention qualify as adverse reactions. |

### 12.4 Recording and reporting of AEs and ARs

All AEs will be recorded within the app and reported to the Chief Investigator within 1 working day (24 working hours). If there is uncertainty that an AE event may reflect an AR, it will be reported to the Trial Steering Committee (TSC) and DMEC and if considered related to either of the apps, will be classified as an AR and reported to the trial Sponsor. who will escalate to the regulatory authority when considered appropriate.

In line with other complex intervention studies, we will monitor non-serious AEs and ARs that are not trial, or treatment related, serious deterioration, and active withdrawals from treatments. All occasions where the score on the PHQ-9 Q9 increases from 0/1 to 2/3 will be separately recorded. The reporting period for all events and reactions will be from first usage of the app until 10 weeks follow-up.

### 12.5 Responsibilities

Researchers at UNIEXE will check for AEs and ARs when participants complete treatment or at follow-up, potentially in response to automated feedback from the website.

Chief Investigator (CI) is responsible via liaison with research team to ensure all AEs are:

1. Recorded within 24 working hours/1 working day of becoming aware of the event and providing further follow-up information as soon as available.
2. Chased with the research team if a record of receipt is not recorded within 2 working days of initial reporting.
3. Distributed to the TSC for consideration if it meets criteria for an AR.
4. Disseminated to the Sponsor and Ethics committee where it is considered the AE may represent an AR.

Sponsor (NB where relevant these can be delegated to CI) is responsible for:

1. Central data monitoring and verification of ARs according to the trial protocol.
2. Reporting safety information to the PI, delegate or independent clinical reviewer for the ongoing assessment of the risk/benefit according to the Trial Monitoring Plan.
3. Reporting AE/ARs that are related to the trial and unexpected, by email to the research ethics committee.
4. Reporting safety information to the independent oversight committees identified for the trial (Data Monitoring and Ethics Committee (DMEC) and/or Trial Steering Committee (TSC)) according to the Trial Monitoring Plan.
5. Preparing standard tables and other relevant information in collaboration with the CI and ensuring timely submission to the MHRA and REC.

### 12.6 Reporting urgent safety measures

If any urgent safety measures are taken, the CI shall immediately, and in any event no later than 3 days from the date the measures are taken, give written notice to the REC, Sponsor and other appropriate bodies where relevant (e.g. MHRA) of the measures taken and the circumstances giving rise to those measures.

### 13. RISK MANAGEMENT

### 13.1 Risk assessment and reporting

Prior to study inclusion we explicitly ask and screen for symptoms of distress, wellbeing and poor mental health (including symptoms of depression, suicidal risk). Whether meeting exclusion criteria at screening or indicating risk at follow up, participants will be provided with automatic feedback with suggested sources of help such as the recommendation to consult their GP or weblinks or phone numbers for national NHS services or those relevant to the Armed Forces Veterans Community.

### 13.1.1 Risk management during baseline assessment

The second screening (i.e., baseline assessment) will allow those with elevated risk to access help pages linking to sources of support as soon as possible. Participants are given the following automated paragraph and options:

**University of Exeter Qualtrics page automated message:**

This study aims to examine the potential for effectiveness of a mobile phone application adapted for female Armed Force Veterans for the management of depression or generalised anxiety. If you are currently struggling with suicidal thoughts or traumatic experiences the trial would not be right for you and specialist help would be the best option. If these issues are affecting you now then we would urge you to reach out to family, friends, or your family doctor/general practitioner. Click the relevant button below for additional links to relevant helpful information and support services available to you. Signposting information detailed earlier will be provided with contact information.

### 13.1.2 Risk management in trial

Once the participant joins the study and starts to use either of the apps, risk is managed by the participant selecting the SOS button or being directed to the SOS button in the event that either PHQ-9 has increased >1 or score has increased >20. Each occurrence of elevated symptomatology (PHQ-9>20) or risk (PHQ-9-Q9>1) when using either app will be logged by the electronic system, the Lead Researcher notified and recorded as an AE.

We note that the nature of the high level of confidentiality means that we do not have relevant GP or family doctor details for participants. The default response is direct information disclosure to the participant of a potential clinical issue with signposting information provided. Transmission of the collected information to the GP will not be possible and requesting this information a priori would negate the ethical and recruitment benefits of pseudo-anonymity and confidentiality for participants. Based on recent recommendations, our default policy is to inform participants of the possibility of them being at risk and to recommend they seek help. This is to prioritise their welfare.

### 13.2 Preventing abuse of participants and risk analysis

Our risk analysis indicates potential theoretical risks and opportunities for abuse of the research findings and of participants within the study including psychological harms (e.g., distress), invasion of privacy (e.g., intrusion into private affairs, public disclosure of embarrassing private information, publicity that puts the individual in a false light to the public, or appropriation of an individual’s name or picture for personal/commercial advantage), loss of confidentiality (personal data becoming public through error or thorough deliberate hacking), and social harms (e.g., embarrassment, stigmatisation). The risk analysis indicates that the likelihood of these risks occurring is relatively low although any potential impact for participants would be high, and, as such, we will enact a detailed participant risk register and update it regularly through the project. Multiple steps and processes will be put into place to mitigate and minimise these risks including (a) explaining potential risks in the information sheet; (b) the welfare procedures described above to minimise participant distress; (c) high levels of security and the use of privacy by design protocols for the app and database; (d) a privacy impact assessment; (e) the emphasis on confidentiality in the project and the separation of collected data from personal identifiers; (f) the use of a code of conduct for all researchers.

In this trial no medical care is provided regardless of the level of risk presented. This is because this trial is for people seeking to manage low mood or excessive worry in a self-help intervention for people with no current risk or have experienced a traumatic episode in the last month. Participants are advised of this at the beginning of the respective app, on the information sheets and sign the section on the Consent Form to confirm that they understand this.

### 13.3 Identifying suicide risk

These are 4 ways within the study that a participant could indicate risk; (1) prior to consent; (2) prior to using the app; (3) end of treatment; (4) follow-up assessment. However, as part of the routine functioning of the Iona EXSW or Iona SHAM CONTROL during use where PHQ-9 increases above 1.

### 13.4 University of Exeter Qualtrics page at second screening (as baseline), 6 and 10 week follow up

A recruit may indicate risk on the website during the baseline assessment or 6 weeks treatment end or 10 weeks follow up in response to questions about suicidality. If the participant scores 2 or 3 on the PHQ-9 Q9, the participant will be encouraged to seek support from family or friends, GP or signposting information to NHS or female Armed Forces signposting information will be provided.

PHQ-9-Q9

Over the last two weeks, how often have you been bothered by any of the following problems? Thoughts that you would be better off dead, or of hurting yourself in some way?

Those scores would represent the answers several days (1), more than half the days (2) and nearly every day (3)

If the potential participant answers yes to either more than half the days (2) or nearly every day (3) to either question, then they would be removed from the trial and automatically presented with the following risk screen:

**University of Exeter Qualtrics page suicide risk automated message:**

**“Your responses to these questions suggest you have been thinking about suicide or about hurting yourself.**

These kinds of thoughts can vary a lot. This may have just been a brief passing thought or reflect a sense of feeling trapped, but without any intention to do anything. These thoughts are relatively common and not that unusual in people who feel down or stressed. If you would like support with these thoughts, please contact your GP or relevant medical professional.

**If you feel at high risk to yourself or others, please contact your GP immediately.**

**You can contact your GP** using the normal telephone number for your GP practice. If the surgery is not open, you will either be automatically redirected to the out-of-hours GP service, or you will be given another number to call. You can also **phone 111 to access the NHS 111 service**, which provides access to local NHS healthcare services in England, and is available 24 hours a day, 365 days a year.

However, you may have been thinking about your death a lot, having persistent thoughts about killing yourself, experiencing suicidal intentions and urges, or be making plans to end your life. In any of these cases or if you have any other thoughts of suicide, we strongly recommend that you contact your general practitioner or family doctor **RIGHT AWAY** for advice and tell them how you are feeling

**If you don't think you can stay safe**, please go to the nearest hospital accident and emergency room. If none of these options are available, please contact a family member or a trusted friend, so that you won't be alone right now. It’s important to seek out the company of people who can support you and who will help to keep you safe.

Try to commit to a plan of action that does not involve suicide. If you have items that may be dangerous for you at home, please consider giving them to a trusted friend, neighbour, the police or a pharmacist for safekeeping until you feel stronger. Try to minimise the use of alcohol or illicit drugs, as using these substances are likely to make your recovery harder. It can also be helpful to think about your faith, loved ones, family and pets. It is important to remember that these feelings and urges do pass, and when individuals feel better, they are glad that they did not act on them. There are effective treatments that can help, and there is no need to struggle alone. Talking to people who understand can make it much easier to manage your symptoms so do please call one of the specialist helplines above. There may be reasons for hope that you have yet to consider. Sometimes the smallest reasons for living can get you through a difficult time. Having thoughts of suicide is nothing to be ashamed of and we encourage you to seek help.

Because the study is focused on managing excessive worry or low mood rather than treating current difficulties, this study is not suitable for you. The Iona mobile phone app has not been designed to help with these difficulties so we are sorry to say that taking part in the study would not be in your best interests at this time. Thank you for your interest.

**We strongly recommend contacting your GP or family doctor as the best person to decide what help you need**.

In addition to your GP or if you don’t feel that you can talk to your GP, there are many useful services and useful sources of support.

We hope that you find one or more of the following helpful:

- **Emergency Services** If you need help and it’s an emergency call: 999
- **NHS 111** If you need help now and its not an emergency call: 111
- **Op Courage** An NHS mental health specialist service designed to help serving personnel due to leave the military, reservists, armed forces veterans and their families. Contact: [mevs.mhm@nhs.net](mailto:mevs.mhm@nhs.net) Call: 0300 034 9986
- **Papyrus 0800 068 4141 or text: 07786 209697** offers National support to young people up to age 35 who are feeling suicidal. (Monday-Friday 10:00am-5:00pm and 7:00pm-10:00pm; 2:00pm-5:00pm on weekends, [pat@papyrus-uk.org](mailto:pat@papyrus-uk.org)
- The **Samaritans** **08457 90 90 90**  Freephone (UK and Republic of Ireland): 116 123 (24 hours) offer a confidential service so you can talk about your feelings, you can contact them at [www.samaritans.org](http://www.samaritans.org), Email: [jo@samaritans.org](mailto:jo@samaritans.org)
- **SANE** offers support to anyone coping with mental illness, including concerned relatives or friends. The SANE helpline **0845 767 8000** is available 7 days a week from 6.pm-11 pm
- **Maytree** is a registered charity supporting people in suicidal crisis and is open for calls and emails 24 hours a day. **– 020 7263 7070,** [maytree@maytree.org.uk](https://www.bipolaruk.org/)
- There are a series of NHS self-help guides which can be found here <https://web.ntw.nhs.uk/selfhelp/>
- There are more guides and online courses here: <https://www.cci.health.wa.gov.au/Resources/Looking-After-Yourself>

In the event a participant indicates suicide risk at end of treatment or 6 week follow up (10 weeks) and this screen is displayed, an automated report/record from the website will be sent to the Lead Researcher and logged by them. The record will show the trial number and the answers to the 3 risk questions. This data will monitor frequency of suicidality across the trial arms.

### 13.5 For participants reporting significant levels of depression at any of the follow-up assessments (defined as PHQ-9 score >20)

The follow-up website will automatically provide them with the following information:

**Follow-up Website Automated message for depression:**

**“Your responses to these questions suggest that within the last month, your overall mood has been low for at least 2 weeks and has had a negative effect on your life. It may be that you are currently experiencing an episode of depression or going through a period of stress or loss.**

If you currently are having problems with the symptoms of depression then **we strongly recommend that you talk to your general practitioner, family doctor or a mental health professional** about your difficulties, as he or she may be able to find ways to help you to improve your mood and handle life’s difficulties better.

If you have not had a health check recently that may also be worth doing so. If you have a diagnosis of depression, please make sure that you follow your treatment regime and consult with the medical professionals involved in your care.

**You can contact your GP** using the normal telephone number for your GP practice. If the surgery is not open, you will either be redirected automatically to the out-of-hours GP service, or you will be given another number to call. You can also **phone 111 to access the NHS 111 service**, which provides access to local NHS healthcare services in England, and is available 24 hours a day, 365 days a year.

As well as your GP, there are many other services available who are really experienced at helping people with your symptoms:

Here are some useful websites that you access directly:

- The **Samaritans** **08457 90 90 90**  Freephone (UK and Republic of Ireland): 116 123 (24 hours) offer a confidential service so you can talk about your feelings, you can contact them at [www.samaritans.org](http://www.samaritans.org),
- There are a series of NHS self-help guides which can be found here <https://web.ntw.nhs.uk/selfhelp/>
- There are more guides and online courses here: <https://www.cci.health.wa.gov.au/Resources/Looking-After-Yourself>
- **Mind** The Mental Health Charity provide information, advice, and support to empower anyone experiencing a mental health problem. They provide information about mental health problems and potential treatments as well as tips for everyday living. [https://www.mind.org.uk/](mailto:jo@samaritans.org)
  - **For info about depression:** <https://www.mind.org.uk/information-support/types-of-mental-health-problems/depression/#.XGQRn1X7SUk>
  - **For apps to help with your mental health and wellbeing:** [https://www.mindcharity.co.uk/advice-information/how-to-look-after-your-mental-health/apps-for-wellbeing-and-mental-health/](https://www.rethink.org/media/1020652/ResourceFinal.pdf)
- **Rethink Mental Illness** Provide expert advice and information to everyone affected by mental health problems, and provide services and groups; [https://www.rethink.org/living-with-mental-illness/](https://www.mindcharity.co.uk/advice-information/how-to-look-after-your-mental-health/apps-for-wellbeing-and-mental-health/)
- **There are a series of NHS self-help guides which can be found here** [https://web.ntw.nhs.uk/selfhelp/](https://youngminds.org.uk/find-help/)
- **There are more guides and online courses here:** <https://www.cci.health.wa.gov.au/Resources/Looking-After-Yourself>

**Helplines**

Alternatively, here are helplines you can ring to talk to someone about what you’re going through:

We hope that you find one or more of the following helpful:

- **NHS 111** If you need help now and it’s not an emergency call: 111
- **Op Courage** An NHS mental health specialist service designed to help serving personnel due to leave the military, reservists, armed forces veterans and their families. Contact: [mevs.mhm@nhs.net](mailto:mevs.mhm@nhs.net) Call: 0300 034 9986
- **Papyrus 0800 068 4141 or text: 07786 209697** offers National support to young people up to age 35 who are feeling suicidal. (Monday-Friday 10:00am-5:00pm and 7:00pm-10:00pm; 2:00pm-5:00pm on weekends, [pat@papyrus-uk.org](mailto:pat@papyrus-uk.org)
- The **Samaritans** **08457 90 90 90**  Freephone (UK and Republic of Ireland): 116 123 (24 hours) offer a confidential service so you can talk about your feelings, you can contact them at [www.samaritans.org](http://www.samaritans.org), Email: [jo@samaritans.org](mailto:jo@samaritans.org)
- **SANE** offers support to anyone coping with mental illness, including concerned relatives or friends. The SANE helpline **0845 767 8000** is available 7 days a week from 6.pm-11 pm
- **Maytree** is a registered charity supporting people in suicidal crisis and is open for calls and emails 24 hours a day. **– 020 7263 7070,** [maytree@maytree.org.uk](https://www.bipolaruk.org/)
- **Papyrus 0800 068 4141 or text: 07786 209697** offers National support to young people up to age 35 who are feeling suicidal. (Monday-Friday 10:00am-5:00pm and 7:00pm-10:00pm; 2:00pm-5:00pm on weekends, [pat@papyrus-uk.org](mailto:pat@papyrus-uk.org)
- The **Samaritans** **08457 90 90 90**  Freephone (UK and Republic of Ireland): 116 123 (24 hours),offer a confidential service so you can talk about your feelings, you can contact them at [www.samaritans.org](mailto:maytree@maytree.org.uk), Email: [jo@samaritans.org](https://www.studentsagainstdepression.org/)
- **SANE** offers support to anyone coping with mental illness, including concerned relatives or friends. The SANE helpline **0845 767 8000** is available 7 days a week from 6.pm-11 pm
- **Maytree** is a registered charity supporting people in suicidal crisis and is open for calls and emails 24 hours a day. **– 020 7263 7070,** [maytree@maytree.org.uk](https://www.mind.org.uk/information-support/types-of-mental-health-problems/bipolar-disorder/about-bipolar-disorder/)
- **The Mix**, Freephone: 0808 808 4994 (13:00-23:00 daily), If you're under 25 you can talk to The Mix for free on the phone, by email or on their webchat. You can also use their phone counselling service, or get more information on support services you might need. [www.themix.org.uk](http://www.themix.org.uk/)

Talking to people who understand can make it much easier to manage your symptoms so **please do call your GP or one of the specialist helplines above**.

### 13.6 Exclusions- Bipolar and Psychosis

If a potential participant is excluded on the basis of a self-reporting prior to giving Consent a previous diagnosis of bipolar disorder or psychosis then would be automatically provided with the following information:

**University of Exeter Qualtrics page automated message for bi-polar disorder:**

“**You have reported that you have previously received a diagnosis of either bipolar disorder or psychosis”.**

Because this study is focused on supporting you to manage excessive worries or low mood the current study is not suitable for you at this time. The Iona mobile phone app has not been designed to help with these difficulties so we are sorry to say that taking part in the study would not be in your best interests at this time. Thank you for your interest.

**Your GP or relevant medical professional is the best person to decide what help you need.**

**You can contact your GP** using the normal telephone number for your GP practice. If the surgery is not open, you will either be re-directed automatically to the out-of-hours GP service, or you will be given another number to call. You can also **phone 111 to access the NHS 111 service**, which provides access to local NHS healthcare services in England, and is available 24 hours a day, 365 days a year.

Alongside your GP, there are other services available to you to provide information and support:

Useful **WEBSITES** that you can access directly below include:

- **Mind** The Mental Health Charity provide information, advice, and support to empower anyone experiencing a mental health problem. They provide information about mental health problems and potential treatments as well as tips for everyday living. [https://www.mind.org.uk/](https://www.studentsagainstdepression.org/)
  - **For bipolar disorder:** [https://www.mind.org.uk/information-support/types-of-mental-health-problems/bipolar-disorder/about-bipolar-disorder/?o=1142#.XGQJJVX7SUk](https://www.rethink.org/living-with-mental-illness/young-people?o=1142#.XGQJJVX7SUk)
  - **For psychosis:** [https://www.mind.org.uk/information-support/types-of-mental-health-problems/psychosis/#.XGQI_lX7SUk](https://www.rethink.org/living-with-mental-illness/young-people#.XGQI_lX7SUk)
  - **For schizophrenia**: <https://www.mind.org.uk/information-support/types-of-mental-health-problems/schizophrenia/about-schizophrenia/?o=6266#.XGQJQlX7SUk>
  - **For apps to help with your wellbeing and mental health:** [https://www.mindcharity.co.uk/advice-information/how-to-look-after-your-mental-health/apps-for-wellbeing-and-mental-health/](https://web.ntw.nhs.uk/selfhelp/)
- **BipolarUK** National charity dedicated to supporting individuals with bipolar, their families and carers. Their websites has information leaflets and links to support, including a peer support line [https://www.bipolaruk.org/](http://www.samaritans.org)
- **Rethink Mental Illness**. Provide expert advice and information to everyone affected by mental health problems, and provide services and groups; including resources specific to young people [https://www.rethink.org/living-with-mental-illness/young-people](http://www.samaritans.org)
  - **Toolkit for young people with questions or worries about their mental health:** [https://www.rethink.org/media/1020652/ResourceFinal.pdf](http://www.samaritans.org)
- **There are a series of NHS self-help guides which can be found here** <https://web.ntw.nhs.uk/selfhelp/>
- **There are more guides and online courses here:** <https://www.cci.health.wa.gov.au/Resources/Looking-After-Yourself>

Alternatively, here are **HELPLINES** you can ring to talk to someone about what how you are feeling

- **Papyrus 0800 068 4141 or text: 07786 209697** offers National support to young people up to age 35 who are feeling suicidal. (Monday-Friday 10:00am-5:00pm and 7:00pm-10:00pm; 2:00pm-5:00pm on weekends, [pat@papyrus-uk.org](https://www.mind.org.uk/information-support/types-of-mental-health-problems/psychosis/)
- The **Samaritans** **08457 90 90 90**  Freephone (UK and Republic of Ireland): 116 123 (24 hours),offer a confidential service so you can talk about your feelings, you can contact them at [www.samaritans.org](https://exeter.content.minddistrict.com/contentresources/0e83f1018dfc42778b169c48491b6de3/resources/out_of_hours_leaflet_uk.pdf), Email: [jo@samaritans.org](https://youngminds.org.uk/find-help/)
- **SANE** offers support to anyone coping with mental illness, including concerned relatives or friends. The SANE helpline **0845 767 8000** is available 7 days a week from 6.pm-11 pm
- **The Mix**, Freephone: 0808 808 4994 (13:00-23:00 daily), If you're under 25 you can talk to The Mix for free on the phone, by email or on their webchat. You can also use their phone counselling service or get more information on support services you might need. [www.themix.org.uk](https://www.rethink.org/media/1020652/ResourceFinal.pdf)

If you find your symptoms particularly distressing or have thoughts about ending your life then please go to the nearest emergency room, or immediately contact your GP.

Talking to people who understand can make it much easier to manage your symptoms so **please do call your GP or one of the specialist helplines above**.

### 14. DISSEMINATION POLICY

There is an overall dissemination policy for the project, within which there is a specific dissemination policy for the trial results, however aspects of the policy may not be applicable depending on the outcome of this Phase II feasibility trial.

Key aspects of the dissemination policy for the trial include:

(i) Consort Guidelines and checklist are reviewed prior to generating any publications for the trial to ensure they meet the standards required for submission to high quality peer reviewed journals etc. <http://www.consort-statement.org/>

(ii) Anonymised data arising from the trial is owned by UNEXE as trial lead and Sponsor.

(iii) All potential publication plans will be reviewed by the Project Steering Committee before release of data to determine appropriate authorship and avoid duplication and replication of effort.

(iv) Authorship will be determined on standard criteria (i.e., consistent with the criteria for individually named authors or group authorship such as The International Committee of Medical Journal Editors defined authorship criteria for manuscripts submitted for publication http://www.icmje.org/recommendations/browse/roles-and-responsibilities/defining-the-role-of-authors-and-contributors.html#two) and will require contributions with respect to design of the study, development of paradigms and interventions within the study, involvement in delivery of the trial, data analysis and/or writing up of the paper. Seniority of authorship will be determined by relative contribution on these elements – individuals leading on design, analysis and write-up of papers will have lead authorship, with this typically following pre-allocated lead roles for the work packages in the grant in the first instance, unless deferred. All papers will include a detailed statement of the relevant author contributions following a standard template.

(v) All publications will acknowledge the funder.

(vi) At the end of the study, a report will be produced and shared with participants who expressed an interest in receiving a copy.

### 15. REFERENCES

Farrand, P., Jeffs, A., Bloomfield, T., Greenberg, N., Watkins, E., & Mullan, E. (2018). Mental health service acceptability for the armed forces veteran community. Occupational Medicine, 68(6), 391-398.

Farrand, P., Mullan, E., Rayson, K., Engelbrecht, A., Mead, K., & Greenberg, N. (2019). Adapting CBT to treat depression in Armed Forces Veterans: qualitative study. Behavioural and Cognitive Psychotherapy, 47(5), 530-540.

Hoffmann, T. C., Glasziou, P. P., Boutron, I., Milne, R., Perera, R., Moher, D., ... & Michie, S. (2014). Better reporting of interventions: template for intervention description and replication (TIDieR) checklist and guide. British Medical Journal, 348.

Jones, N., Jones, M., Greenberg, N., Phillips, A., Simms, A., & Wessely, S. (2020). UK military women: mental health, military service and occupational adjustment. Occupational Medicine, 70(4), 235-242.

Kroenke, K., Spitzer, R. L., & Williams, J. B. (2001). The PHQ‐9: validity of a brief depression severity measure. Journal of General Internal Medicine, 16(9), 606-613.

Leung, L. B., Rubenstein, L. V., Post, E. P., Trivedi, R. B., Hamilton, A. B., Yoon, J., ... & Yano, E. M. (2020). Association of veterans affairs primary care mental health integration with care access among men and women veterans. JAMA Network Open, 3(10), e2020955-e2020955.

Luik, A. I., Bostock, S., Chisnall, L., Kyle, S. D., Lidbetter, N., Baldwin, N., & Espie, C. A. (2017). Treating depression and anxiety with digital cognitive behavioural therapy for insomnia: a real world NHS evaluation using standardized outcome measures. Behavioural and Cognitive Psychotherapy, 45(1), 91-96.

Mundt, J. C., Marks, I. M., Shear, M. K., & Greist, J. M. (2002). The Work and Social Adjustment Scale: a simple measure of impairment in functioning. The British Journal of Psychiatry, 180(5), 461-464.

National Institute for Health and Care Excellence. (2022). Depression in adults: treatment and management [NG222]. London: NICE.

National Institute for Health and Care Excellence. (2020). Generalised anxiety disorder and panic disorder in adults: management. Clinical guideline [CG113]. London: NICE.

Parkes, S., Croak, B., Brooks, S. K., Stevelink, S. A., Leightley, D., Fear, N. T., ... & Greenberg, N. (2023). Evaluating a smartphone app (MeT4VeT) to support the mental health of UK armed forces veterans: feasibility randomized controlled trial. JMIR Mental Health, 10(1), e46508.

Spitzer, R. L., Kroenke, K., Williams, J. B., & Löwe, B. (2006). A brief measure for assessing generalized anxiety disorder: the GAD-7. Archives of Internal Medicine, 166(10), 1092-1097.

Zhou, L., Bao, J., Setiawan, I. M. A., Saptono, A., & Parmanto, B. (2019). The mHealth app usability questionnaire (MAUQ): development and validation study. JMIR mHealth and uHealth, 7(4), e11500.

### APPENDIX

Appendix 1: Feasibility Outcomes

| **Feasibility Outcomes** | | | | |
| --- | --- | --- | --- | --- |
| Number of people accessed study web site | |  | | |
| Number of people consented | |  | Report by demographics | |
| Number of people randomised | |  | Report by demographics | |
| Blinding randomisation maintained | | Yes | | No |
| Study arms | |  | | |
| Iona FV | Number downloaded app |  | | |
|  | Number completed baseline outcome measure | Data completion of each item for each outcome measure | | |
|  | Number completed treatment |  | | |
|  | Number completed end of treatment outcome measures | Data completion of each item for each outcome measure | | |
|  | Number completed final outcome measures 10 week post treatment start | Data completion of each item for each outcome measure | | |
| Iona SHAM CONTROL | Number downloaded app |  | | |
|  | Number completed baseline outcome measure | Data completion of each item for each outcome measure | | |
|  | Number completed treatment |  | | |
|  | Number completed outcome measures at 6 weeks post baseline | Data completion of each item for each outcome measure | | |
|  | Number completed final outcome measures 10 week post treatment start | Data completion of each item for each outcome measure | | |

Appendix 2: Outcome Measures

| 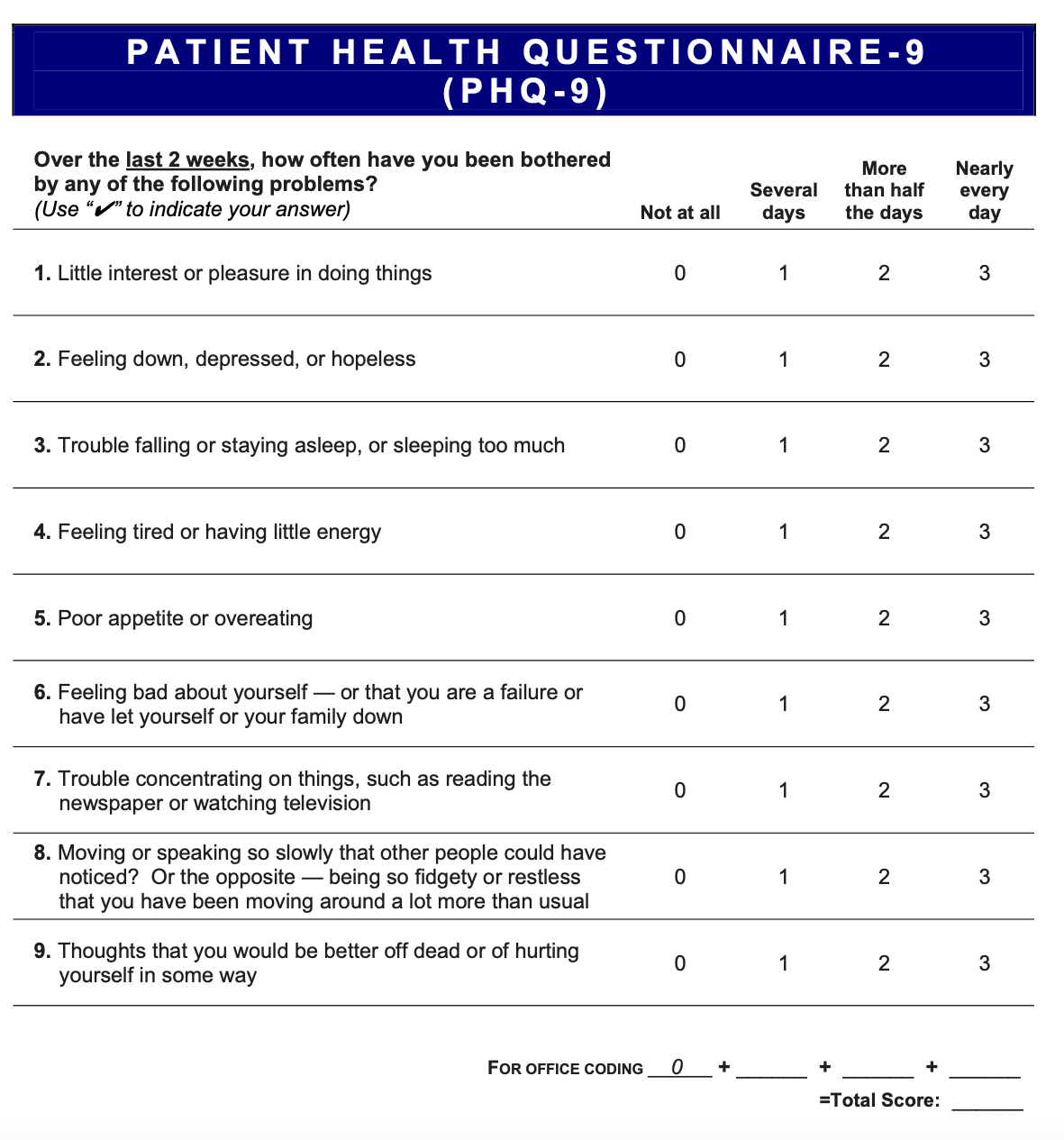 |
| --- |

| 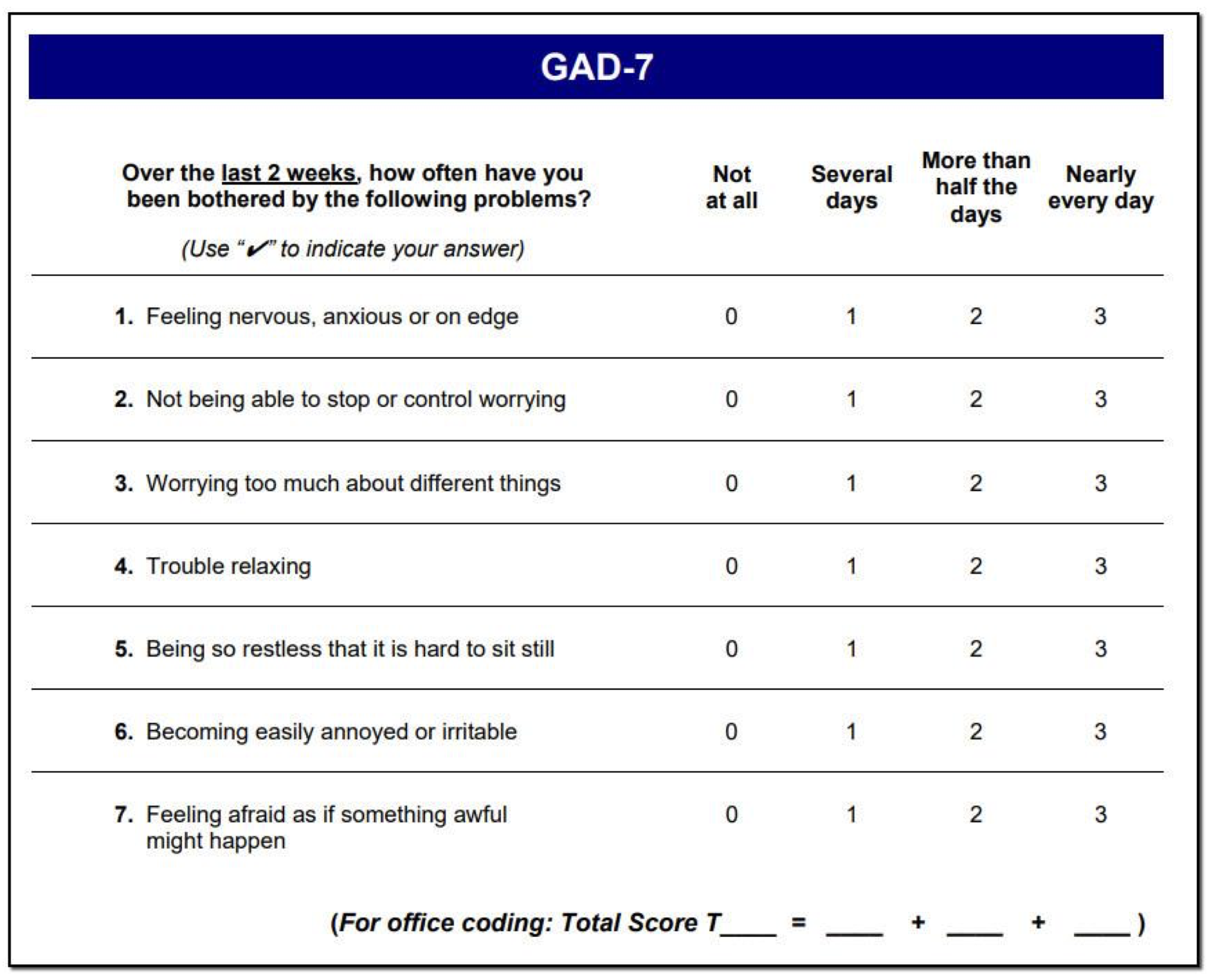 |
| --- |

| 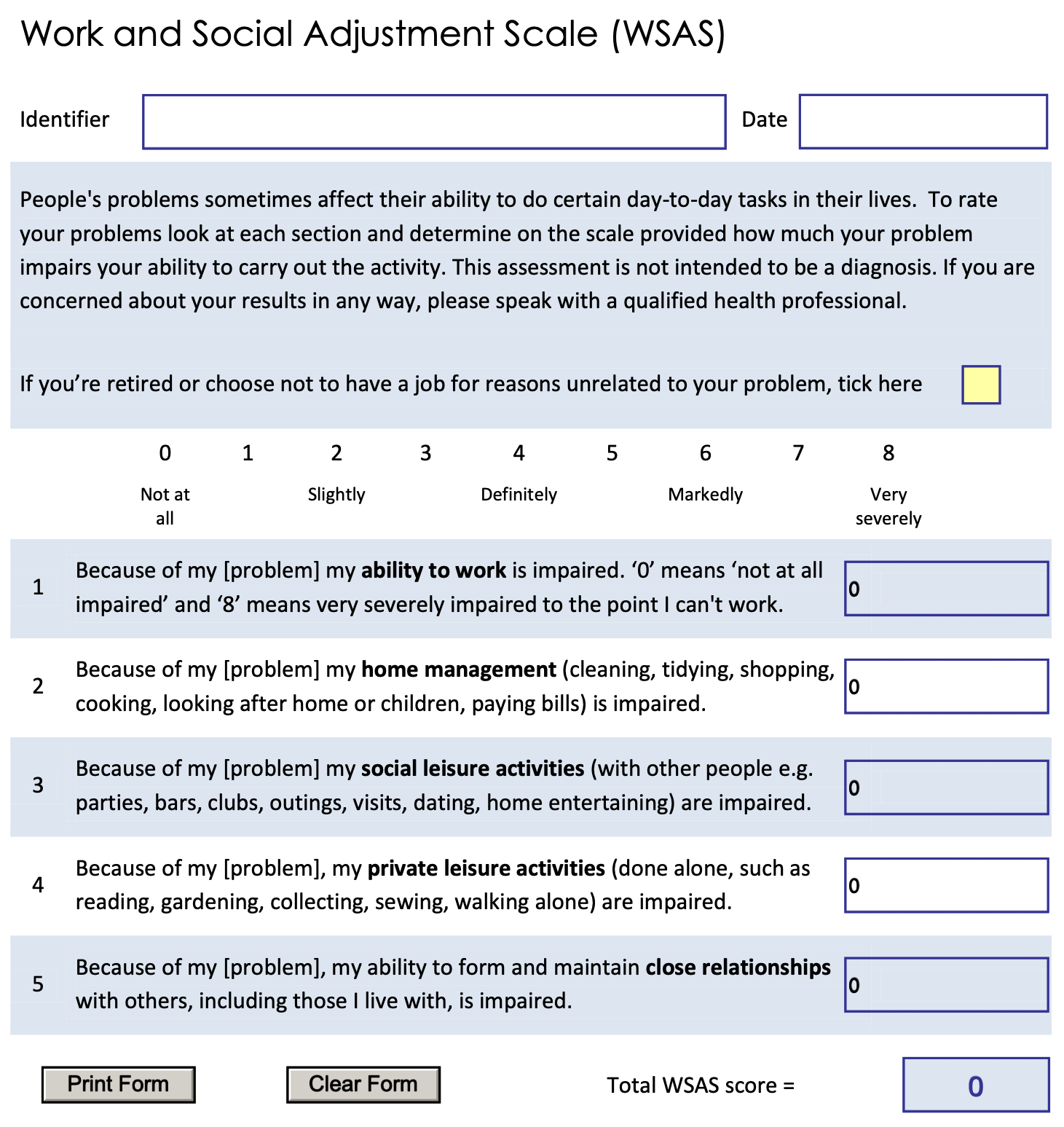 |
| --- |

Mobile Acceptability and Usability Questionnaire (MAUQ)


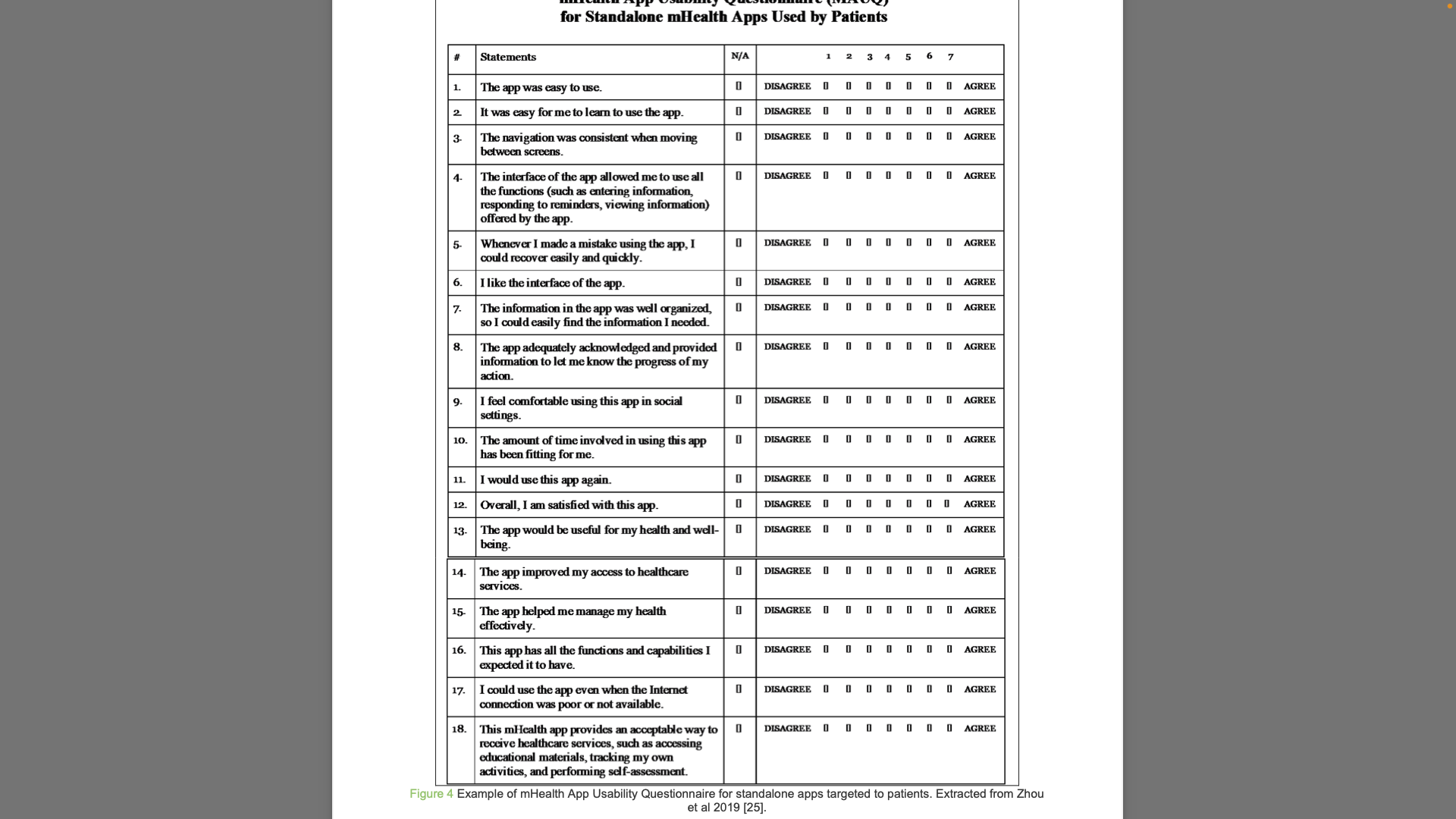

Supplement: online supplemental file 1 [file bmjopen-16-3-s001.docx]
