# Supplementary material for: Cognitive–behavioural therapy smartphone app for low mood and worry management in female armed forces veterans in Great Britain: protocol for a feasibility randomised controlled trial
Source: BMJ Open. 2026 Mar 6;16(3):e112494. doi: 10.1136/bmjopen-2025-112494 (PMC12970052; doi:10.1136/bmjopen-2025-112494)
Supplement: Supplementary file 2 [file bmjopen-2025-112494.reviewer_comments.pdf]

PEER REVIEW HISTORY

BMJ Open publishes all reviews undertaken for accepted manuscripts. Reviewers are asked to complete a checklist review form and are provided with free text boxes to elaborate on their assessment. These free text comments are reproduced below.

ARTICLE DETAILS

Title (Provisional)

Protocol for a Feasibility Randomised Controlled Trial: Cognitive Behavioural Therapy Smartphone app for Low Mood and Worry Management in Female Armed Forces Veterans in Great Britain

Authors

Janbakhsh, Melika; Turnbull, Elizabeth; Baker, Jonathan; Bacon, Andy; Farrand, Paul

---

VERSION 1 - REVIEW

**Reviewer**	**1**
**Name**	**Leightley, Daniel**
**Affiliation**	**King's College London**
**Date**	**01-Dec-2025**
**COI**	**None**

This protocol outlines a feasibility randomised controlled trial of a digital intervention designed to support the management of low mood and worry among women who have served in the UK Armed Forces. The topic is important and the focus on a population often underrepresented in research is welcome. It is also great to see a push in the digital space for Defence. However, recruitment has already been completed and follow-up is partly underway. This limits the scope for methodological refinement, so my comments focus on clarity, transparency, and accurate reporting of what was done. Also to prepare for future work submissions; where reviewers may raise these concerns.

It would be helpful if the authors clearly state that recruitment was complete at the time of protocol submission and confirm that the manuscript reflects the ethics-approved protocol. If any deviations occurred, these should be described plainly.

Overall.

* There is a conceptual inconsistency around how the app is framed. Early sections refer to a "wellbeing app", while later sections describe a tool to support management of low mood and worry using CBT principles. These are not synonymous and the distinction should be

clarified. This is important when the manuscript states the app is for the management of low mood CBT stye which does give indication of a medical app.

* The protocol refers to MRC guidance for pilot RCTs, yet the study is described as a feasibility trial. The terms feasibility and pilot have distinct meanings in MRC guidance, so it would be helpful to explain why one label was chosen over the other.

Introduction.

* The introduction would benefit from a broader description of digital mental health activity within Defence. Beyond MeT4VeT, there are several evaluated digital tools in the Armed Forces context and the rationale for an app specifically tailored to women who have served should be articulated more clearly.

* Please add a definition of “veteran” for international readers.

* Please provide a definition for veteran for international readers.

* The reference to “imagery” and its relevance to Defence (line 68) would benefit from a short explanation.

Methods.

* Recruitment channels are now reported, but given recruitment is complete, the authors could describe all avenues actually used.

* While a feasibility study, it would be advisable for a clearer description of adverse event (AE) and serious adverse event (SAE) procedures, including definitions, reporting pathways, and responsibilities.

* Eligibility criteria points on antidepressant medication and mental health support would benefit from clarification. The distinction between being on stable antidepressant medication and receiving active mental health treatment should be explained, along with the rationale for allowing one but excluding the other.

* The description of how the app was adapted for women who have served lacks practical detail. It would help to specify what changes were made (e.g. language, imagery, content emphasis, modules).

* The methods appears to suggest some form of PPIE/coproduction but it is not clear how this operated in reality. Was it just one meeting, approval changes, or did the authors use an agile approach to development?

* In relation to the app itself. I have several comments.

* Please provide screenshots of the app interface, it is difficult to ascertain what it actually is.

* Was the app a native app or a progressive web app?

* Was the app publicly available or did participants download it via Apple TestFlight/Google Test?

* The use of the term wellbeing for apps is often used by developers; can you confirm if the app was listed in the app store under wellbeing OR medical.

* Please report if push notifications were used; if so, how often, and what causes the trigger.

* I assume internet is also inclusive of Wi-Fi.

* The write up appears to suggest that the sham was not “validated’ and by extension perhaps not even tested. Can you confirm it was at leats tested in some participants prior to undertaking the feasibility study?

* I query the metric of success being used as “2 days” within 6 weeks would count as an engaged user. This is not in keeping with the literature, and not in keeping with wider app norms. A clear rationale for this metric is required and supported by the literature.

* It is not clear what the primary outcome measure is; and what is actually the measure of success. Citing Table 3 as the outcome measures is confusing, and many of the “successes” appear unscientific and lack rationale. Please provide a simple measure of success, such as usability scores (e.g. greater than 5 demonstrates that the app is acceptable to the target population).

* For example, the suggestion that engaged users of 20% as a progression criteria (measure of success) is acceptable, the authors should provide some citations to justify this.

* I don’t understand the sample size calculation. Is this for the feasibility or the main study. I am unable to recreate this myself. Please provide all the factors required to compute a power calculation.

* The description of the sham app would benefit from a clearer explanation of how it controls for expectancy effects. At present, it appears to differ quite substantially in content and depth.

* I query the suggestion that this was double blind RCT. The lead author has declared an interest in Iona Mind which is the developer of the app. They are part of the research team. Can you confirm that they themselves were blinded? It is perfectly acceptable to state that the developer is part of the research team and not blinded. But this should be reported and it would become a single-blinded RCT.

* How was actual blinding for participants done? Was it based on the QR code then allowing access to the SHAM, or was it two apps with different names?

* Data collection and management only mentions Qualtrics data; however, the outcome measures also include app data. This therefore should be amended to include how app data was managed (e.g. module completions).

* Please outline the trial management structure (e.g. project management group, trial steering committee) and procedures for protocol amendments.

Results.

* None reported.

Discussion.

* No comments.

Misc.

* If the funder had the opportunity to review or approve the manuscript before submission, please outline steps taken to minimise bias and whether the funder requested changes.

* It would help to specify whether the intervention is intended to sit within an NHS IAPT-style stepped-care model, or whether it is purely standalone self-help. This matters for feasibility markers and for how the results will be interpreted.

VERSION 1 - AUTHOR RESPONSE

**Reviewer's comments**		
**Daniel Leightley**		
Intro		
It would be helpful if the authors clearly state that recruitment was complete at the time of protocol submission and confirm that the manuscript reflects the ethics-approved protocol. If any deviations occurred, these should be described plainly.	Thank you for your feedback. We have amended this:<br>*For clarity, recruitment had concluded at the time this protocol paper was submitted. Additionally, this manuscript reflects the final protocol approved by ethics.*	164 - 166
There is a conceptual inconsistency around how the app is framed. Early sections refer to a "wellbeing app",	We agree that the categorisation of apps is currently unclear and apologise for the discrepancy in the language that gives rise to the conceptual inconsistency. We have now addressed this by ensuring amendments consistent with previous papers exploring the base Iona app.<br>Farrand, P., Raue, P. J., Ward, E., Repper, D., Baker, J., & Areán, P. (2024). Use and engagement with low-intensity cognitive behavioral therapy techniques used within an app to	

while later sections describe a tool to support management of low mood and worry using CBT principles. These are not synonymous and the distinction should be clarified. This is important when the manuscript states the app is for the management of low mood CBT stye which does give indication of a medical app.	support worry management: content analysis of log data. *JMIR mHealth and uHealth*, *12*, e47321.<br>Farrand, P., Raue, P. J., Ward, E., Healey, S., & Baker, J. (2025). Low-intensity behavioral activation smartphone app to support low mood: description, engagement and log data analysis. *Academia Mental Health and Well-Being*, *2*(2).<br><br>Specifically, language is consistent and does not refer to treatment but 'support' and that IonaFFV, and the prior non adapted Iona app, are wellbeing approaches. This is made clear in the in the TIDieR app description app where it is stated:<br>*.. provided with the signposting information and reminded that IonaFFV is not intended to deliver treatment and should not be used outside of the context of a wellbeing self-help aid.*<br>The app status as a Wellbeing app is supported by the inclusion in the text that addresses another comment regarding listing. The base Iona app and IonaFFV have only ever been listed under 'Wellbeing' on the respective app stores.<br>Furthermore, we have also removed reference to depression and Generalised Anxiety Disorder which are indeed diagnostic categories and used non diagnostic terms to reflect non-diagnosis inclusion criteria. The app is not intended to be used for treatment but as a 'wellbeing' device, and this is made clear during onboarding within the app.	Line 261–point 4
The protocol refers to MRC guidance for pilot RCTs, yet the study is described as a feasibility trial. The terms feasibility and pilot have distinct meanings in MRC guidance, so it would be helpful to explain why one label was chosen over the other.	Thank you for raising this important point, it was an unfortunate oversight on our behalf. We have removed mention of "pilot" with respect to this study throughout the paper and replaced it with "feasibility". We have reinforced this important distinction at the end of the Discussion.<br>*Should the progression criteria be met, results may be used to inform the design of a Phase II pilot RCT. Insights from the pilot could then guide modifications to design improvements for a Phase III definitive trial to evaluate the intervention's effectiveness in improving low mood and reducing worry among female forces veterans. If progression criteria are not met, the intervention and design of the feasibility study could be revised to address identified limitations prior to further evaluation.*	551-557
The introduction would benefit from a broader description of digital mental health activity within Defence. Beyond MeT4VeT, there are several	We have now elaborated on the use of digital mental health approaches within defence and cited several developments. Furthermore, we explore limitations associated with these developments explicitly for female force veterans which justifies the development of IonaFFV and subsequent need to focus on evaluation.	95-114

evaluated digital tools in the Armed Forces context and the rationale for an app specifically tailored to women who have served should be articulated more clearly.		
Method		
* The reference to "imagery" and its relevance to Defence (line 68) would benefit from a short explanation.	A short explanation has been added:<br>*However, factors such as in-service adversity[3], language, and male-veteran-focused branding contribute to the perception that existing services primarily reflect the needs of male veterans[4]. Alongside limited awareness of available support options, these can create additional barriers for female veterans seeking mental health support[5]. As a result, female veterans may feel more reluctant to access current services available for armed forces veterans and are therefore less likely to seek help[6].*	71-76
Please add a definition of "veteran" for international readers.	A short definition has been added:<br>*Both male and female forces veterans, a person who has served in the armed forces for at least one day[1], experience barriers accessing mental health treatment[2].*	69-70
Recruitment channels are now reported, but given recruitment is complete, the authors could describe all avenues actually used.	This has been updated:<br>*Recruitment is primarily conducted online through public and private Armed Forces community Facebook groups, Armed Forces veterans' charity and community sector organisations' social media posts, and Armed Forces social media platforms, such as LinkedIn and Facebook, and word of mouth. Armed Forces Facebook pages will be contacted to seek permission to post the study flyer on their page.*	371-375
While a feasibility study, it would be advisable for a clearer description of adverse event (AE) and serious adverse event (SAE) procedures, including definitions, reporting	Thank you very much for your comment. This information has been added to the manuscript:<br><br>*Adverse Events (AE)*<br>*For the purposes of this study, an adverse event (AE) is defined as any deterioration in a participant's mental state or behaviour following the administration of the approach. This includes events that may not be directly caused by or related to the intervention. An AE is flagged when a participant's score on item 9 (i.e., suicidal ideation screening) of the PHQ-9 increases to 2 (more than half the days) or 3 (nearly every day) or the participant's overall PHQ-9 deteriorates from 'mild' or 'moderate' to 'severe' during the week six and week ten*	461-482

pathways, and responsibilities.	*follow ups. AEs will be monitored and recorded by the research team (MJ and ET) within one working day, after which they will provide follow-up information as soon as possible.*<br>*Adverse Reaction (AR)*<br>*An adverse reaction (AR) is defined similarly to an AE, however, requires establishing a reasonable possibility that the event is related to the app.*<br>*Responsibilities*<br>*PF, as the Chief Investigator, is responsible for reporting to the TSC for consideration when an event meets criteria for an AR, and for disseminating to the Sponsor and Ethics committee where the AE is considered to represent a potential AR.*<br>*Serious Adverse Events (SAE)*<br>*Recording SAE is not possible in this study, as no mental-health or community-based services are involved.*	
Eligibility criteria points on antidepressant medication and mental health support would benefit from clarification. The distinction between being on stable antidepressant medication and receiving active mental health treatment should be explained, along with the rationale for allowing one but excluding the other.	We have made the following amendment:<br><br>Inclusion criteria 7<br>*To preserve internal validity, participants who have started or changed antidepressant medication within the past month will be excluded from the study to minimise instability of symptoms and concurrent active intervention effects*[37].<br><br>Inclusion criteria 8<br>*To avoid active co-intervention effects, participants currently receiving mental health support will be excluded, as concurrent psychological support can influence outcomes targeted by liCBT and obscure the attribution of outcomes to the intervention*[37].	220-222<br><br>224-227
The description of how the app was adapted for women who have served lacks practical detail. It would help to specify what changes were made (e.g.	Thank you for your comment, we added below information to the manuscript:<br><br>*More specifically, after consultation with a panel of female armed forces veterans and a wider armed forces stakeholder group, the armed forces-specific context, such as label and graphics, were adapted to better suit the target audience. For example, the label 'ex-servicewomen' was changed to 'female armed forces veterans' to better represent female veterans. These were done through a 5-phase process*[19]*. This resulted in*	135-141

language, imagery, content emphasis, modules).	*the development of Iona for female forces veterans (IonaFFV), demonstrated to have good levels of acceptability, usability and utility*[19].	
The methods appears to suggest some form of PPIE/ coproduction but it is not clear how this operated in reality. Was it just one meeting, approval changes, or did the authors use an agile approach to development?	Further information is added to the Patient and Public Involvement and Engagement (PPIE) section<br>*A PPIE group, comprising five female Armed Forces veterans and a wider armed forces stakeholder panel with expertise across the Armed Forces community was involved in adapting the IonaFFV app*[19]*. The wider stakeholder group met once to discuss the app's adaptation and development, focusing on tailoring it to meet the specific needs of female forces veterans and producing an initial version. This version was subsequently reviewed in a focus group with the female armed forces to identify potential areas for improvement Based on the feedback, further adaptations were made, and a second focus group with the same panel was convened to review the revisions*[19].<br>*The PPIE group also contributed to the design of the feasibility study protocol. Additionally, a Trial Steering Committee (TSC) comprising Armed Forces stakeholders and a female veteran from the adaptation process was established to work in partnership with the research team throughout the study. Furthermore, a website will be developed in collaboration with the PPIE group upon study completion to facilitate dissemination of the findings.*	177-191
Please provide screenshots of the app interface, it is difficult to ascertain what it actually is	Added to the intervention section.<br>*Iona FFV and Iona sham app interfaces*<br>*Both Iona FFV and sham app share general features in their interface. Figure 3 captures some of the Iona app's interface features, which are representative of some of the app's features that were adapted during the development process*[19]*. Iona app graphics uses the tri-service colours to emphasise the association with the armed forces (Figure 4, A). Additionally, at the start of the app several quotes are shown. These quotes are a mixture of those from female forces veterans as well as more generally acknowledging the transition from service in the armed forces to civilian life (Figure 4, B). Further, logo and names of trusted organisations are provided at the start of the app to enhance perceived credibility and expectancy of effectiveness of the app (Figure 4, C). Finally, a map is provided in the app to indicate other users' location, which enhances engagement with the app (Figure 4, D).*	280-293

	Figure 4. Screenshots of Iona FFV and Iona sham app	
Was the app a native app or a progressive web app?	Thank you for your comment. We have included the below statement:<br>*IonaFFV is a native digital mobile phone-based approach to support low mood and worry management and is consistent with the characteristics associated with liCBT self-help[10].*	239-245
The use of the term wellbeing for apps is often used by developers; can you confirm if the app was listed in the app store under wellbeing OR medical.	We confirm IonaFFV has only ever been listed in the app store under 'Wellbeing'. This is further specified in Section 4 of the TIDieR Checklist and again on line 261.<br><br>And again, on line 395.	261<br><br>395
Was the app publicly available or did participants download it via Apple TestFlight/Google Test?	We have added more information to clarify how participants accessed to download the app:<br>*All participants are asked to download the same app. The app is available under the 'Wellbeing' listing on Apple store and Google play to download. To log in and use the app, participants will be asked to enter their unique code which then brings them to either the Iona FFV or the Iona Sham version of the app, whichever one they are randomised to.*	394-398
Please report if push notifications were used; if so, how often, and what causes the trigger. Again this relates to develop0ment paper!	This is now addressed in the TIDieR.<br>*To maintain engagement, participants receive at least one push notification per day. These notifications include reminders to engage with scheduled activities, mark activities as completed in the app, and complete modules.*	261, point 4 - And 278, point 4
I assume internet is also inclusive of Wi-Fi.	The term internet was also used to include Wi-Fi but we have now included this where internet is highlighted.	
The write up appears to suggest that the sham was not "validated' and by extension perhaps not even tested. Can you confirm it was at least tested in some participants	We have made this clear.<br>*Prior to use, the sham was beta tested but not validated[19]*	269

prior to undertaking the feasibility study?		
I query the metric of success being used as “2 days” within 6 weeks would count as an engaged user. This is not in keeping with the literature, and not in keeping with wider app norms. A clear rationale for this metric is required and supported by the literature.	Thank you for your comment. After careful consideration, we have now defined an engaged user based on available evidence from the literature. Based on the systematic review by Fleming et al. (2018), we have added the following definition to the manuscript:<br>*Therefore, for the purposes of this study, an engaged user is defined as a user who completes at least two educational modules (i.e., lessons) and a minimum of two sessions during the six-week period of using the Iona app. Specifying a minimum engagement of two lessons and two sessions is consistent with existing literature suggesting that brief or partial exposure is common and may be sufficient for feasibility assessment in early stage digital interventions.*	323-328
It is not clear what the primary outcome measure is; and what is actually the measure of success. Citing Table 3 as the outcome measures is confusing, and many of the “successes” appear unscientific and lack rationale. Please provide a simple measure of success, such as usability scores (e.g. greater than 5 demonstrates that the app is acceptable to the target population).	Thank you for your comment. There is not a universally agreed threshold for the MAUQ to define acceptable score. However, as this is a Likert-scale rating from 1 (strongly disagree) to 7 (strongly agree), with 4 labelled as neutral, it is generally agreed to recognise scores above 4 as good useability.<br><br>Below text has been added to the manuscript:<br>*Further, although there is no universally agreed threshold to define an acceptable MAUQ score, responses of strongly agree, agree, and somewhat agree are considered acceptable by some researchers*[66,67].	329-331
For example, the suggestion that engaged users of 20% as a progression	We clarified our rationale for progression criteria below:<br>*These thresholds for progression criteria are set as feasibility objectives rather than as clinical assumptions or a measure of success or effectiveness of the intervention. Recent methodological guidance on progression criteria for an*	310-322

criteria (measure of success) is acceptable, the authors should provide some citations to justify this.	*external feasibility pilot trial recommends that thresholds need to be interpreted as guidelines rather than prescriptive rules and be context-specific and aligned with feasibility objectives[62].*<br>*This is particularly important given there are no universally accepted engagement benchmarks for engagement, and that engagement with mental health and app in real-world setting is generally low, with the majority of users disengaging after downloading the app[64]. Evidence from a systematic review of real-world engagement with digital self-help intervention indicated that between 21% and 88% of users achieved at least minimal engagement defined as using the intervention at least once or completing one module or one assessment[65].*	
I don't understand the sample size calculation. Is this for the feasibility or the main study. I am unable to recreate this myself. Please provide all the factors required to compute a power calculation.	Thank you for your comment. Information on sample size has been updated in the manuscript:<br>*With a total sample size of 60, a 70% retention rate at the 4-week follow up can be estimated with a margin of error of approximately ±12% using a 95% confidence interval or ±10% using a 90% confidence interval.*	363-366
I query the suggestion that this was double blind RCT. The lead author has declared an interest in Iona Mind which is the developer of the app. They are part of the research team. Can you confirm that they themselves were blinded? It is perfectly acceptable to state that the developer is part of the research team and not blinded. But this should be reported and it	We argue that the study is double blind. As PF was engaged with Iona Mind at the time the study was conducted, we ensured he remained unaware as to study arm and did not have contact to the data. This is made clear<br>*PF will not be involved in any data collection or data analysis. Data collection and analysis will be solely done by the study researchers (MJ and ET).*<br><br>This is reiterated in the "Conflict of Interest" section:<br>*PF will be blinded throughout the study.*<br><br>Furthermore, to ensure the data reflected the interpretation to minimise all bias the DMC will be asked to approve the results section draft in the subsequent paper.<br>*Prior to unblinding, the DMC will review a draft of the results section that employs coded identifiers to represent each trial arm. This review will evaluate the accuracy, clarity, and consistency of data reporting, with study researchers only becoming unblinded once the draft has been reviewed and approved by the DMC.*	423-425<br><br>578-579<br><br>500-504

would become a single-blinded RCT.		
How was actual blinding for participants done? Was it based on the QR code then allowing access to the SHAM, or was it two apps with different names?	Thank you for your comment. We have now amendment the manuscript. All participants downloaded the same app but were provided with a unique code. After entering the unique code, they were directed to either the Iona FFV or Iona Sham control.<br><br>*All participants are asked to download the same app. The app is available under the 'Wellbeing' listing on Apple store and Google play to download. To log in and use the app, participants will be asked to enter their unique code which then brings them to either the Iona FFV or the Iona Sham version of the app, whichever one they are randomised to.*<br><br>And how the code directed participants to different apps:<br>*Following randomisation, participants will be sent a link to download the app. Once the app is downloaded, participants will be asked to input their unique code. Codes from one group will open the Iona FFV version of the app onto a participant's phone, and codes from the other group will open the Iona sham app.*<br><br>We also explained in more details how the double blinding was implemented:<br>*Participants are given a unique code from either group A or group B, which corresponds to the Iona FFV app or the Iona sham app. Neither the researchers nor the participants are aware of which group contained the codes for which app.*	394-398<br><br>415-418<br><br>404-407
Data collection and management only mentions Qualtrics data; however, the outcome measures also include app data. This therefore should be amended to include how app data was managed (e.g. module completions).	This has been addressed.<br>*Only data related to the feasibility objectives collected through the University of Exeter Qualtrics system will be analysed. Researchers will not have any access to the in-app data collected, such as GAD-7 and PHQ-9 and these will not be used in any data analysis. Research data collected through the University of Exeter Qualtrics will be analysed. Log data will be managed by Iona Mind and will be shared with, and analysed by, the researchers to determine engagement with the app across the two groups.*	200-206
Please outline the trial management structure (e.g. project	We have now added this detail.<br>*The trial is overseen by a Trial Management Group led by the Chief Investigator and Lead Researcher, who are responsible for the day-to-day conduct, data monitoring, protocol*	435-442

management group, trial steering committee) and procedures for protocol amendments.	*adherence and safety reporting. This is alongside the TSC who provide independent oversight. As the Trial Sponsor, the University Sponsorship Committee, ensures compliance with General Data Protection Regulation, Good Clinical Practice and ethic approvals. All amendments to the protocols are logged and reported to the TSC and Trial Sponsor, and where substantive changes required, to the Ethics Committee.*	
If the funder had the opportunity to review or approve the manuscript before submission, please outline steps taken to minimise bias and whether the funder requested changes.	The funders (Office for Veterans Affairs – Cabinet office), have had the opportunity to review the manuscript prior to submission, but solely to ensure no misrepresentation regarding their involvement and to ensure appropriate acknowledgement. If after acceptance but before publication they request further amendments these will be fully discussed with the Editor.	
It would help to specify whether the intervention is intended to sit within an NHS IAPT-style stepped-care model, or whether it is purely standalone self-help. This matters for feasibility markers and for how the results will be interpreted.	We have now made this clear in the Introduction<br><br>*As a standalone approach, it may be particularly well suited for adoption within services currently provided for armed forces veterans, with the potential to address existing reluctance to engage[6]. However, because it is designed to be used independently, it would not be suitable for integration within NHS Talking Therapies for anxiety and depression[25].*	124-128

VERSION 2 - REVIEW

**Reviewer**	**1**
**Name**	**Leightley, Daniel**
**Affiliation**	**King's College London**
**Date**	**05-Feb-2026**
**COI**	

The authors have addressed my comments.

I do however not agree that this is a true double blind study. It is participant blinded, not double blinded. It is perfectly acceptable to have developers as part of the research team; but they do have access to the data, and are able to identify allocation patterns. Therefore, it is not a true double blinded study as we would have with a clinical trials teams.
